# Recent Developments in Stereoselective Reactions of Sulfoxonium Ylides

**DOI:** 10.3390/molecules30030655

**Published:** 2025-02-01

**Authors:** Ciarán O’Shaughnessy, Mukulesh Mondal, Nessan J. Kerrigan

**Affiliations:** 1 School of Chemical Sciences, Dublin City University, Glasnevin, D09 V209 Dublin, Ireland; ciaran.oshaughnessy9@mail.dcu.ie; 2 Department of Chemistry, Oakland University, Rochester, MI 40309, USA; mukuleshm@gmail.com

**Keywords:** sulfoxonium ylide, epoxidation, aziridination, cyclopropanation, olefination, insertion, asymmetric synthesis, diastereoselectivity, enantioselectivity

## Abstract

This review probes the recent developments in stereoselective reactions within the area of sulfoxonium ylide chemistry since the early 2000s. An abundance of research has been applied to sulfoxonium ylide chemistry since its emergence in the early 1960s. There has been a continued effort since then with work in traditional areas, such as epoxidation, aziridination and cyclopropanation. Efforts have also been applied in novel areas, such as olefination and insertion reactions, to develop stereoselective methodologies using organocatalysis and transition metal catalysis. The growing research area of interrupted Johnson–Corey–Chaykovsky reactions is also described, whereby unexpected stereoselective cyclopropanation and epoxidation methodologies have been developed. In general, the most observed mechanistic pathway of sulfoxonium ylides is the formal cycloaddition: (2 + 1) (e.g., epoxides, cyclopropanes, aziridines), (3 + 1) (e.g., oxetanes, azetidines), (4 + 1) (e.g., indanones, indolines). This pathway involves the formation of a zwitterionic intermediate through nucleophilic addition of the carbanion to an electrophilic site. An intramolecular cyclization occurs, constructing the cyclic product. Insertion reactions of sulfoxonium ylides to X–H bonds (e.g., X = S, N or P) are also observed, whereby protonation of the carbanion is followed by a nucleophilic addition of X, to form the inserted product.

## 1. Introduction

Sulfur ylides were first discovered in the 1930s by Jessop and Ingold, and the further use and exploration of sulfur ylides through the synthesis of epoxides was initially observed by A.W. Johnson, discovering the interesting methylene transfer properties of sulfonium ylides to aldehydes [1,2]. This was further probed by Corey and Chaykovsky in the 1960s through the use of both sulfonium and sulfoxonium ylides in reaction with aldehydes and ketones [3,4,5]. Sulfur ylides can be divided into two categories, depending on the oxidation number of the sulfur: sulfonium ylides and sulfoxonium ylides. The neighboring carbanion of a sulfonium ylide is a better nucleophile due to the instability of the ylide in comparison to its sulfoxonium ylide counterpart, which is more stable due to stabilization by delocalization in addition to electrostatic interactions. However, sulfoxonium ylides have more efficient leaving group ability through the sulfoxide group’s propensity to leave. In terms of structural properties contributing to stability, sulfur ylides with electron-withdrawing groups adjacent to the carbanion are described as ‘stabilized sulfur ylides’. The most common sulfoxonium ylide used in synthesis, dimethylsulfoxonium methylide, **2**, is unstabilized, as there are no deactivating groups adjacent to the carbanion in addition to the sulfoxonium group. When comparing unstabilized sulfoxonium ylides and unstabilized sulfonium ylides, Corey and Chaykovsky discovered the former to be stable at room temperature in THF for several days, while the latter decomposed under the same conditions after a few minutes [5].

**Scheme 1:**
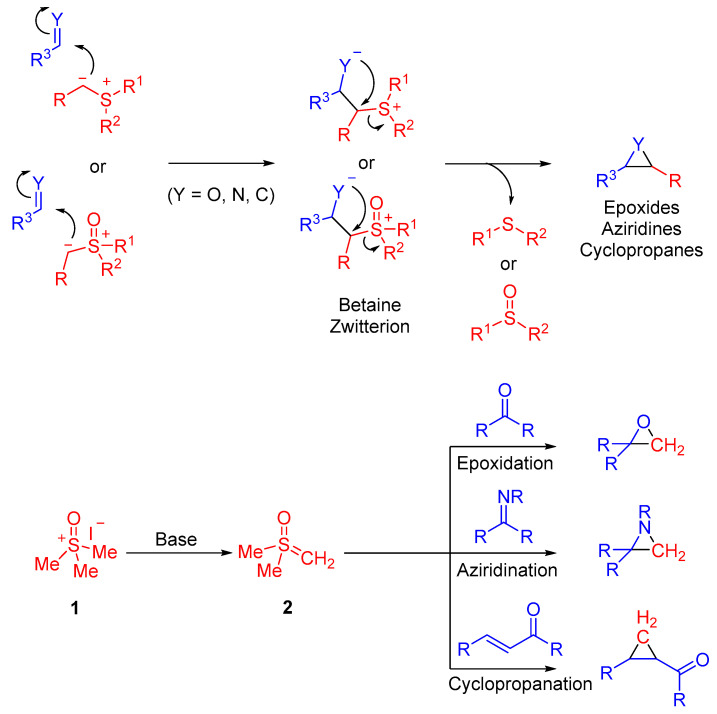
Epoxidation, aziridination and cyclopropanation reactions are the most common applications of the Johnson–Corey–Chaykovsky reaction.

The Johnson–Corey–Chaykovsky reaction pioneered an effective reaction pathway to synthesize three-membered heterocycles and carbocycles (Scheme 1). A sulfur ylide is prepared by deprotonation from the *α*-position of the sulfonium/sulfoxonium salt. Due to the potential of the positively charged sulfonium/sulfoxonium group to stabilize an adjacent carbanion, this increases the acidity of *α*-protons. In general, sulfur ylides react via formal cycloadditions, initially through nucleophilic addition to an electrophile forming a betaine intermediate which intramolecularly cyclizes to form a desired 3-membered heterocycle or carbocycle, with concomitant cleavage of dimethylsulfide or dimethylsulfoxide (DMSO). Therefore, the Johnson–Corey–Chaykovsky reaction transforms aldehydes, ketones, imines and alkenes into epoxides, aziridines and cyclopropanes, respectively, via a methylene transfer from sulfur ylides [6,7,8].

**Scheme 2:**
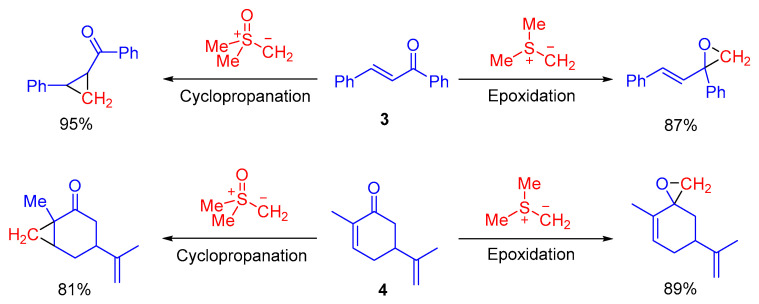
Chemoselectivity comparison of sulfoxonium and sulfonium ylides in reaction with *α*,*β*-unsaturated carbonyl compounds.

Corey and Chaykovsky observed a notable reactivity difference when comparing the methylene transfer pathways of sulfoxonium ylides and sulfonium ylides in reaction with *α*,*β*-unsaturated carbonyl compounds [4,5]. Methylene transfer to a carbon–carbon double bond resulting in a cyclopropane appeared to be favored over the formation of an epoxide. This chemoselectivity switch to favor reaction at the *β*-site (1,4-addition) over the carbonyl site (1,2-addition) initially came as a surprise. However, when probing dimethylsulfonium methylide and dimethylsulfoxonium methylide individually against chalcone, **3**, and against carvone, **4**, it was found that sulfonium ylides favored the epoxidation reaction and sulfoxonium ylides favored cyclopropanation (Scheme 2). Unstable sulfonium ylides prefer methylene transfer to the carbonyl, whereas the more stabilized sulfoxonium ylides enable methylene transfer to the activated alkene (*β*-position). It was also determined that stabilized sulfonium ylides can enable cyclopropanation to occur [5,8,9]. Sulfoxonium ylides are softer nucleophiles than unstabilized sulfonium ylides. As a result, the *α*,*β*-unsaturated carbonyl more readily undergoes 1,4-addition at the alkene double bond (softer electrophilic site), than 1,2-addition at the carbonyl group. In contrast, unstabilized sulfonium ylides are harder nucleophiles than sulfoxonium ylides and will form an epoxide when reacting with a carbonyl group of an *α*,*β*-unsaturated carbonyl.

**Scheme 3:**
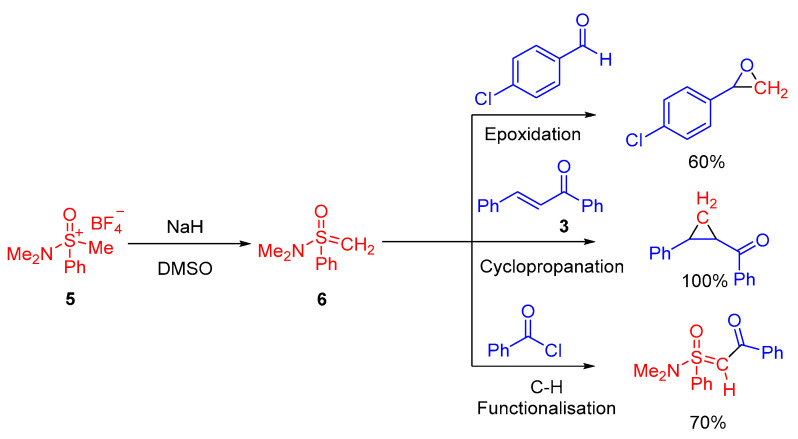
Applications of *α*-aminosulfoxonium ylides carried out by Johnson and colleagues.

In addition to carbon-substituted sulfoxonium ylides, C.R. Johnson and colleagues explored the reactivity of *α*-aminosulfoxonium ylides in reaction with aldehydes, *α*,*β*-unsaturated esters, acyl chlorides and isocyanates [10]. In analogous fashion to the preparation of dimethylsulfoxonium methylide, *α*-aminosulfoxonium ylides can be prepared by deprotonation of the corresponding aminosulfoxonium salts using sodium hydride in DMSO and, in turn, they can be utilized to synthesize desired products, such as epoxides, cyclopropanes and aziridines (Scheme 3). Apart from offering an alternative methodology to synthesize these desired products, *α*-aminosulfoxonium ylides **6** formed from their parent salt **5** are more stable, can be prepared under milder conditions than their carbon-based sulfoxonium and sulfonium ylide analogues, and provide a better leaving group in the sulfinamide (compared to DMSO from sulfoxonium salts). Johnson showed that the epoxidation of *p*-chlorobenzaldehyde proceeded in a reasonable yield (60%) and that the chemoselective cyclopropanation of chalcone **3** went with full conversion (100%). The carbon–hydrogen bond functionalization of *α*-aminosulfoxonium ylides was also exhibited in reaction with benzoyl chloride.

The described review focuses on stereoselective advances in the area of sulfoxonium ylide chemistry over the last decade, elaborating and broadening discussions made in previous reviews and documenting recent progressions [6,7,8].

## 2. Special Characteristics of Sulfoxonium Ylides

**Scheme 4:**
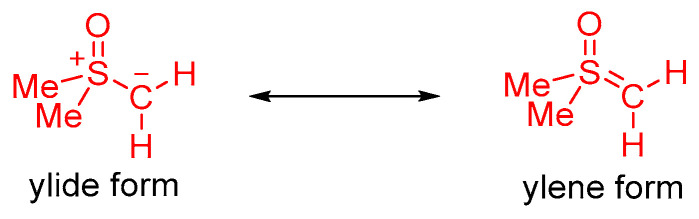
Canonical forms of sulfoxonium ylides: ylide and ylene.

Sulfoxonium ylides are typically drawn in one of two canonical forms: ylide and ylene. The resonance hybrid structures are represented in Scheme 4. The true form of the sulfoxonium ylide exists somewhere between the two [6,7].

The synthetic capabilities of sulfur ylides have been extensively explored over the years due to the excellent leaving group ability of sulfides/sulfoxides allowing for a prompt synthesis of epoxides, aziridines or cyclopropanes as the carbon–sulfur bond is comparatively weak [11,12,13,14,15]. In addition to this desired attribute, sulfur ylides have a relatively high degree of stability when compared to their ammonium ylide or oxonium ylide analogues and hold a comparable stability to phosphorus ylides. Organic chemists have held a strong interest in the chemistry of sulfur and the element’s innate ability to stabilize a neighboring carbanion. The initial theory proposed to explain this stabilizing ability was the potential of the filled lone pair orbital (n) of the carbanion to delocalize into vacant d-orbitals [11,12,13,14,15,16]. However, since computational studies were carried out, the most accepted theory behind the element’s ability to stabilize a neighboring carbanion has been attributed to two factors. The first factor is the electrostatic attraction of the positively charged sulfur atom and the negatively charged carbanion. The second factor that is proposed to create stabilization is the negative hyperconjugation through overlapping orbitals, between the filled lone pair orbital (n) and the σ* orbital of the carbon–sulfur bond [11,12,13,14,15,17,18,19].

Therefore, the ability to stabilize an adjacent carbanion along with the DMSO/sulfinamide functioning as an effective leaving group has allowed sulfoxonium ylides to attract a great deal of attention. Along with stabilizing ability and good leaving group ability, sulfoxonium ylides possess an effective blend of good nucleophilicity coupled with lower basicity when compared to the extreme case of unstabilized carbanions, such as organolithium reagents (e.g., RLi), which are better nucleophiles but also possess a much higher degree of basicity. High basicity frequently leads to unwanted side reactions (e.g., elimination reactions). The pKa values of methyl-substituted sulfonium and sulfoxonium salts are 16–18 in DMSO, whereas for methyl-substituted phosphonium and ammonium salts, the pKa is >20 in DMSO [20,21,22,23]. 3-*Exo-tet* cyclization reactions involving sulfur ylides, such as epoxidation or cyclopropanation, are made possible as a result of sulfides’ and sulfoxides’ general stability/good leaving group ability, in contrast to phosphine and amine leaving groups where these transformations do not readily occur [24].

## 3. Synthesis of Sulfoxonium Ylides

**Scheme 5:**
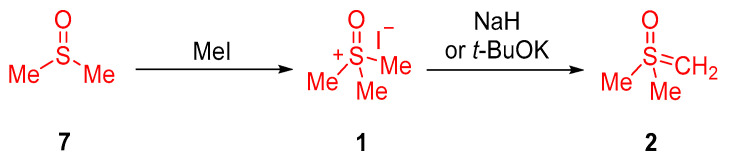
Most common pathway to prepare dimethylsulfoxonium methylide from DMSO.

The most common and recognizable sulfoxonium ylide used in organic synthesis is dimethylsulfoxonium methylide **2**, which is formed by base deprotonation of an acidic *α*-hydrogen of trimethylsulfoxonium iodide **1** [3,4,5]. Through the work of Kuhn and Trischmann, the formation of trimethylsulfoxonium iodide **1** was observed in an insertion reaction (S_N_2) between DMSO **7** and methyl iodide (Scheme 5) [25,26]. The follow-up reaction to prepare the ylide involves the deprotonation of a methyl group to form a carbanion, which is stabilized by the neighboring positively charged sulfoxonium group. This can alternatively be represented as a methylide (ylene form) as discussed earlier (Scheme 4). Dimethylsulfoxonium methylide **2** is considered an ‘unstabilized’ sulfoxonium ylide; however, there have been many methods developed to prepare ‘stabilized’ sulfoxonium ylides. In effect, stabilizing an ylide requires the addition of another deactivating functional group *β* to the sulfoxonium group.

**Scheme 6:**
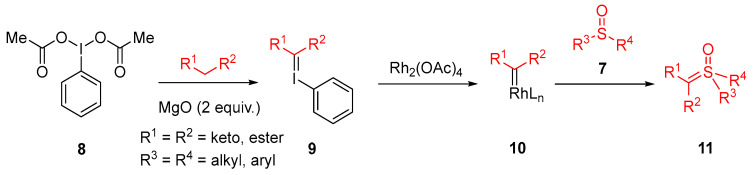
Vaitla’s rhodium-catalyzed synthesis of sulfoxonium ylides via in situ formed iodonium ylides and sulfoxides.

In 2017, Vaitla and colleagues developed a methodology to prepare sulfoxonium ylides with keto and ester groups, which are substituted at the *α*-position from sulfoxides and bis-substituted methanes through rhodium catalysis (Scheme 6) [27]. The reaction proceeds through the in situ formation of an iodonium ylide **9** from iodosobenzene diacetate **8** and the *β*-dicarbonyl substrate under basic conditions. Following this, the iodonium ylide reacts with rhodium(II) acetate to form a rhodium-carbene complex **10**, which reacts with DMSO **7** or a differentially substituted sulfoxide to form a sulfoxonium ylide disubstituted at the *α*-position **11**. The most effective reaction conditions were when the reaction was carried out in a microwave at 100 °C for 5 min, using magnesium oxide in 1,2-dichloroethane. This reaction provided 27 examples with yields of up to 81%, with reasonable substrate scope tolerance [27].

**Scheme 7:**
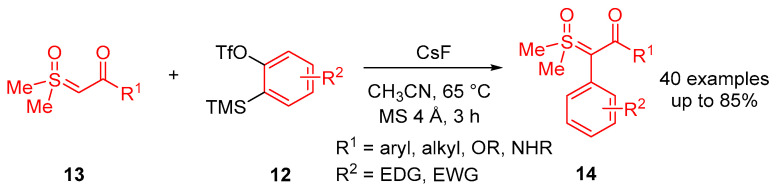
Burtoloso’s synthesis of pro-chiral *α*-aryl-*α*-carbonyl sulfoxonium ylides from the cross-coupling of arenes and sulfoxonium ylides.

Burtoloso and colleagues provided a new synthetic pathway to form pro-chiral *α*-aryl-*α*-carbonyl sulfoxonium ylides **14** using the cross-coupling of arenes **12** and sulfoxonium ylides **13**, with keto, ester or amide groups substituted at the *α*-position of the methylide (Scheme 7) [28]. The optimal conditions to carry out this cross-coupling reaction were found to utilize CsF to aid the formation of benzyne in CH_3_CN using molecular sieves (4 Å) at 65 °C for 3 h, with a molar ratio of 1:1 of arene **12** to sulfoxonium ylide **13**. The mechanism is proposed to proceed via the formation of an aryne through the use of fluoride displacing the trimethylsilyl group and hence promoting triple-bond formation by elimination of the triflate group. Following this, benzyne reacts with the sulfoxonium ylide to form pro-chiral *α*-aryl-*α*-carbonyl sulfoxonium ylides **14**. These new sulfoxonium ylides hold the potential to engage in stereoselective reactions due to this prochirality. This methodology was observed to have a broad substrate scope, displaying 40 examples with reaction yields of up to 85% [28].

**Scheme 8:**
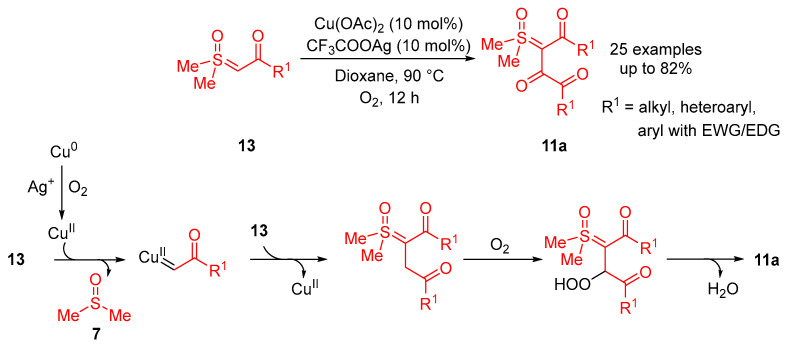
Proposed reaction mechanism of the copper-catalyzed homocoupling of *α*-ketosulfoxonium ylides.

In 2020, Zou and colleagues reported a new methodology to synthesize *α*,*α*,*β*-tricarbonyl sulfoxonium ylides **11a** through a copper-catalyzed homocoupling of *α*-ketosulfoxonium ylides **13** [29]. The proposed reaction mechanism starts with an initial oxidation of the copper catalyst, followed by the formation of a copper carbene intermediate through reaction with an *α*-ketosulfoxonium ylide **13**, with cleavage of DMSO **7**. The carbene intermediate inserts into a second molecule of *α*-ketosulfoxonium ylide, releasing the copper catalyst. The sulfoxonium ylide intermediate undergoes oxidation in the presence of molecular oxygen to form a hydroperoxide group *α* to the ylidic carbon. Following a dehydration step, an *α*,*α*,*β*-tricarbonyl sulfoxonium ylide **11a** is formed (Scheme 8).

The methodology functioned most effectively in dioxane at 90 °C for 12 h under an oxygen atmosphere, using 10 mol% of copper(II) acetate and 10 mol% of silver(I) trifluoroacetate. The novel prochiral sulfoxonium ylides prepared in this methodology hold excellent potential for stereoselective reactions. This paper reported 25 examples with yields of up to 82% and displayed moderate tolerance of aryl electronic variation [29].

**Scheme 9:**
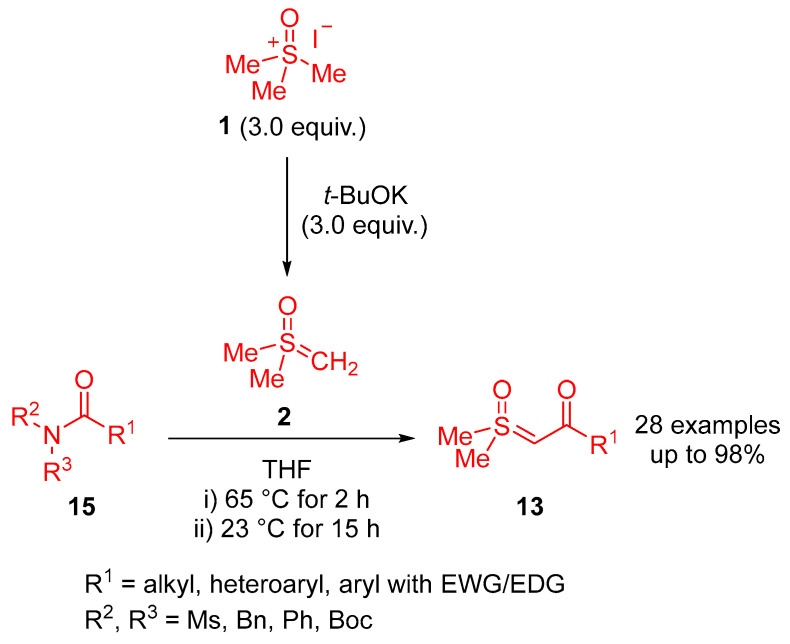
Szostak and Rahman’s synthesis of *α*-ketosulfoxonium ylides from amides and dimethylsulfoxonium methylide.

Szostak and Rahman introduced a methodology to synthesize *α*-ketosulfoxonium ylides **13** from trimethylsulfoxonium iodides **1** and protected amides **15** under basic conditions (Scheme 9) [30]. The reaction proceeds through a selective N–C(O) cleavage by nucleophilic addition of dimethylsulfoxonium methylide **2** to the protected amide (methylide:amide in a 3:1 molar ratio). The optimal reaction conditions were observed to involve THF as solvent, with an initial ylide preparation step at 65 °C for 2 h, followed by 15 h at 23 °C to prepare the *α*-ketosulfoxonium ylide product **13**. The methodology displayed broad scope for the preparation of aryl, heteroaryl and alkyl ketosulfoxonium ylides, with 28 examples in reaction yields of up to 98%.

**Scheme 10:**
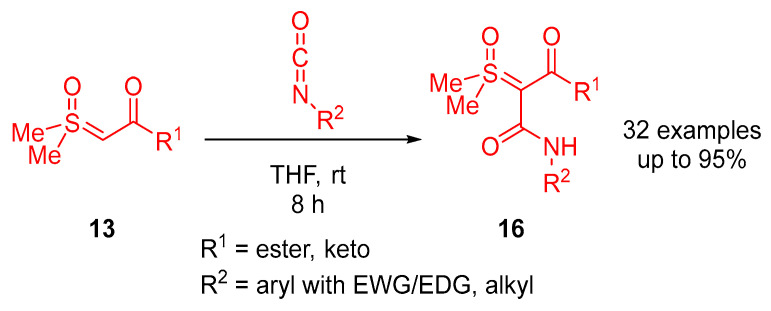
Pandey’s synthesis of *α*-keto-*α*-amide sulfoxonium ylides from isocyanates and sulfoxonium ylides.

In 2023, Pandey and colleagues showed through the reaction of *α*-ketosulfoxonium ylides **13** and isocyanates that *α*-keto-*α*-amide sulfoxonium ylides **16** could be prepared under mild conditions (Scheme 10) [31]. The reaction proceeds through an initial nucleophilic addition of the sulfoxonium ylide to the electrophilic carbon-center of the isocyanate, followed by a proton transfer, which results in amide substitution at the *α*-position. The desired bis-substituted sulfoxonium ylide product **16** holds potential to construct further promising chiral centers. The reaction was found to perform most effectively in THF for 8 h at room temperature under inert conditions. The optimal molar ratio was observed to be a 1.5:1 ratio of isocyanate to sulfoxonium ylide for the most desirable results. The synthesized *α*-keto-*α*-amide sulfoxonium ylides **16** held a wide range of keto and amide functionalization, where alkyl, aryl and heteroaryl groups were found to be tolerated. The reaction showed a broad substrate scope with 32 examples and reaction yields of up to 95% [31].

**Scheme 11:**
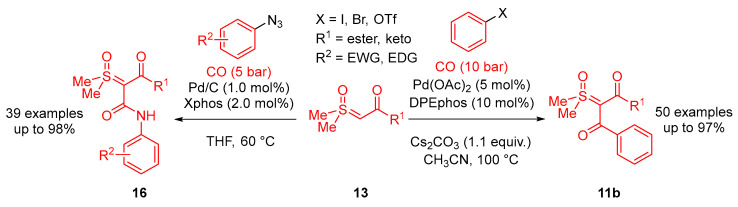
Wu’s palladium-catalyzed synthesis of *α*,*α*-dicarbonylsulfoxonium ylides and *α*-carbonyl-*α*-amidesulfoxonium ylides.

In recent years, chemists have looked to palladium catalysis as a tool to prepare sulfoxonium ylides. Wu and Yuan developed a method to prepare *α*,*α*-dicarbonylsulfoxonium ylides from aryl halides (or aryl triflates) and *α*-ketosulfoxonium ylides (or *α*-estersulfoxonium ylides) (Scheme 11) [32]. The methodology utilizes carbon monoxide (10 bar) as a C1 source for carbonylation. For the palladium-catalyzed carbonylation of *α*-estersulfoxonium ylides, the optimal reaction conditions were reported as employing 5 mol% of palladium(II) acetate, 10 mol% of bis[(2-phenylphosphino)phenyl] ether (DPEphos) and 1.1 equivalents of caesium carbonate in CH_3_CN at 100 °C for 20 h with carbon monoxide. The reaction performed optimally with 2.5 mol% of the palladium catalyst, 5.0 mol% of ligand and at 80 °C when a series of *α*-ketosulfoxonium ylides were probed. This methodology was observed to have a broad substrate scope, displaying 50 examples of **11b** with reaction yields of up to 97%. It, in particular, displayed a wide tolerance for aryl halides and aryl triflates, where both electron-withdrawing and electron-donating groups (EWG/EDG) performed successfully against a wide range of *α*-ester sulfoxonium ylides and *α*-keto sulfoxonium ylides. In 2020, Wu and colleagues developed another palladium-catalyzed carbonylation to synthesize *α*-carbonyl-*α*-amidesulfoxonium ylides from aryl azides and *α*-keto or *α*-estersulfoxonium ylides, showing 39 examples of **16** with reaction yields of up to 98% [33]. In the case, the ligand utilized was dicyclohexyl [2′,4′,6′-tris(propan-2-yl)[1,1′-biphenyl]-2-yl]phosphane (XPhos). The reaction displayed tolerance of activated and deactivated aryl azides with reaction yields in the range of 68–98%.

**Scheme 12:**
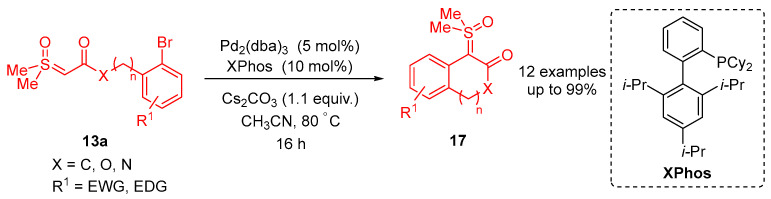
Aïssa’s synthesis of cyclic sulfoxonium ylides using palladium-catalysis.

A methodology to synthesize cyclic sulfoxonium ylides **17** was reported in 2023 by Aïssa and colleagues through the intramolecular arylation of sulfoxonium ylides **13a** (Scheme 12) [34,35,36]. The reaction mechanism was expected to proceed via a palladium-catalyzed coupling of the aryl halide and ylide moieties in the sulfoxonium ylide **13**. The palladium catalyst oxidatively adds or inserts into the Ar–Br bond initially. The cyclic sulfoxonium ylide **17** forms through reductive elimination of a Pd(Ar)(sulfoxonium ylide) intermediate. The best reaction result was observed using 5 mol% of tris(dibenzylideneacetone)dipalladium(0), 10 mol% of XPhos and 1.1 equivalents of caesium carbonate in CH_3_CN in 80 °C for 16 h. The methodology performed successfully for 12 examples with reaction yields of up to 99%, permitting the synthesis of new compounds of substituted benzene fused to *γ*-lactones, *δ*-lactones, *ω*-lactones, cyclopentanones and cyclohexanones [34,35,36].

**Scheme 13:**
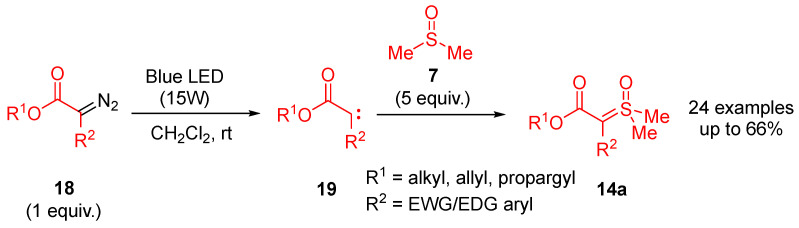
Jurberg’s synthesis of *α*-aryl-*α*-estersulfoxonium ylides using blue-light.

In recent times, the use of visible light in the formation of *α*-estersulfoxonium ylides and *β*-ketosulfoxonium ylides from *α*-diazoesters and *α*-diazoketones, respectively, has been explored with great success. Jurberg and colleagues developed a methodology to synthesize *α*-aryl-*α*-estersulfoxonium ylides **14a** from DMSO **7** and an *α*-estercarbene **19** derived from *α*-diazoester **18** using blue LED light, reporting 24 examples with reaction yields of up to 66% (Scheme 13) [37]. The blue light-induced formation of *α*-aryl-*α*-estersulfoxonium ylides **14a** was observed to perform most optimally in CH_2_Cl_2_ at room temperature under air for 12 h.

**Scheme 14:**
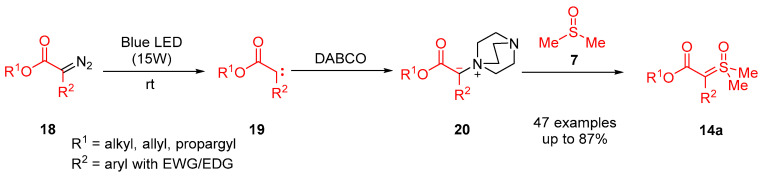
Bhat’s blue-light induced synthesis of *α*-aryl-*α*-estersulfoxonium ylides using DABCO.

In 2021, Bhat and colleagues developed a similar methodology to synthesize *α*-aryl-*α*-estersulfoxonium ylides **14a** with the use of 1,4-diazabicyclo[2.2.2]octane (DABCO) under Ar using blue light (15W) for 14–24 h depending upon the substrate (Scheme 14) [38]. Indeed, 47 examples were reported with reaction yields of up to 87%.

**Scheme 15:**
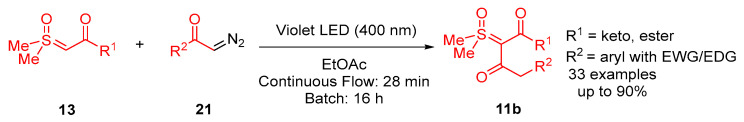
Burtoloso’s violet light-induced synthesis of *α*,*α*-diketosulfoxonium ylides.

In 2022, Burtoloso and colleagues reported a violet light-induced C-H functionalization of *α*-ketosulfoxonium ylides **13** by *α*-diazoketones **21** to form to *α*,*α*-diketosulfoxonium ylides **11b** (Scheme 15) [39]. This method demonstrated a broad substrate scope with 33 examples and reaction yields of up to 90%. The synthesis was developed to perform under both continuous flow (28 min) and batch (16 h) conditions. Both modes functioned using a 15W violet LED (400 nm) in EtOAc. The mechanism proceeds through an initial Wolff rearrangement of *α*-diazoketone **21** to a ketene, which undergoes nucleophilic attack by the *α*-ketosulfoxonium ylide **13**. Subsequently, an acidic *α*-hydrogen undergoes a proton transfer to form the *α*,*α*-diketosulfoxonium ylide **11b**.

## 4. Reactions of Sulfoxonium Ylides

### 4.1. Epoxidation

**Scheme 16:**
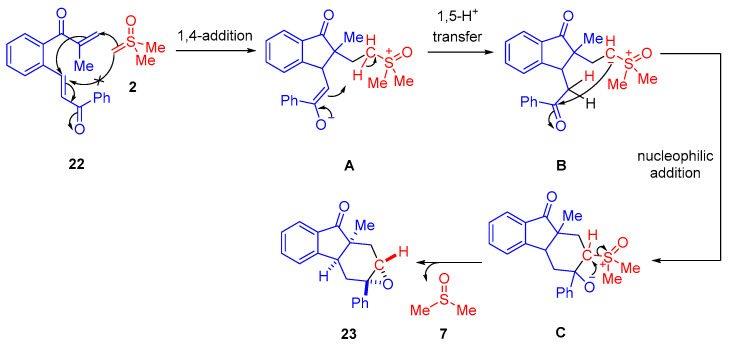
Proposed reaction mechanism of Ramasastry’s interrupted Johnson–Corey–Chaykovsky reaction of dimethylsulfoxonium methylide and tethered bis-enones.

A recent development in stereoselective epoxidation reactions came from an unexpected area of sulfoxonium ylide chemistry. The quickly developing area of interrupted Johnson–Corey–Chaykovsky reactions has seen recent successes in mostly stereoselective cyclopropanation reactions [40,41,42]. However, success has also been observed in stereoselective epoxidation reactions [43]. In 2024, Ramasastry and colleagues developed an interrupted Johnson–Corey–Chaykovsky reaction methodology involving the unexpected formation of 2,3-epoxyhexahydrofluoren-9-ones **23** from tethered bis-enones **22** (Scheme 16) [43]. In the majority of reactions of dimethylsulfoxonium methylide and tethered bis-enone, the expected cyclopropanation of either *α*,*β*-unsaturated ketone group is not observed. The reaction proceeds through the expected 1,4-addition of ylide **2** to the methylene of **22**, as opposed to the competing methine. Another, and unexpected, conjugate addition occurs to the methine resulting in an arene-fused cyclopentanone **A**. The ketone group reforms through a 1,5-H^+^-transfer, resulting in another sulfoxonium ylide **B**. A hexahydrofluorenone **C** is formed through a nucleophilic addition reaction whereby the *α*-carbanion adjacent to the sulfoxonium group acts as a nucleophile to attack the carbonyl group of the ketone. Epoxidation occurs through the quick cleavage of DMSO **7**, resulting in 2,3-epoxyhexahydrofluoren-9-one **23**.

The initial investigation started with optimization of the reaction. They probed the reaction of **22** and dimethylsulfoxonium methylide **2** using different bases and solvent systems at room temperature for 5–10 min. The reaction proceeded best in terms of yield and diastereoselectivity (dr) in DMF using 1.2 equivalents of a base. Bases included NaH (84%, dr 2:1), potassium *t*-butoxide (63%, dr 1.7:1), caesium carbonate (76%, dr 1.7:1) and lithium bis(trimethylsilyl)amide (65%, dr 3.5:1). The reaction proceeded moderately in CH_3_CN using NaH (66%, dr 1.3:1). Poor yield and diastereoselectivity was observed when NaH was tested in THF (19%, dr 1:1) and 1,4-dioxane (22%, dr 1:1) and no reaction was observed when Et_3_N and 1,1,3,3-tetramethylguanidine were explored in DMF. Despite lithium bis(trimethylsilyl)amide enabling the best diastereoselectivity, the combination of NaH in DMF at room temperature was found to be the most effective overall.

**Scheme 17:**
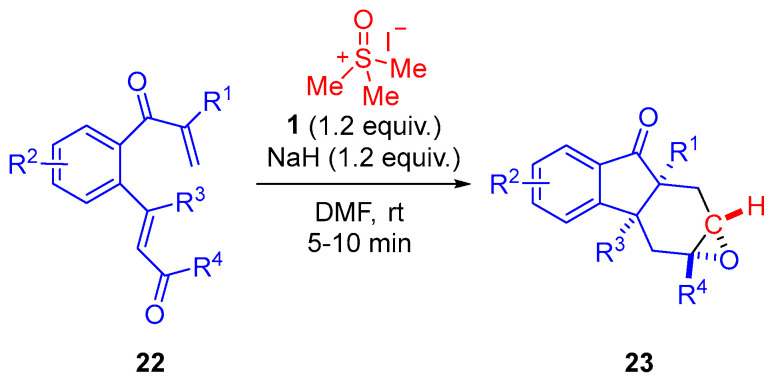
Ramasastry’s diastereoselective epoxidation of bis-enone with dimethylsulfoxonium methylide.

The scope of the reaction was examined, resulting in 23 examples with reaction yields of up to 84% and diastereoselectivity of up to 3:1 (Scheme 17, Table 1). The R^1^ substituent responded well when substituted with alkyl groups and sterically large alkyl groups bonded to phenyl rings. The arene backbone was investigated, the R^2^ substituent was found to be tolerant of naphthalene, activated aryls and deactivated aryls bearing fluorine. The R^4^ substituent performed well where aliphatic ketones and aryl ketones were probed. Both activated and deactivated aryl ketones were tolerated. In most cases, the R^3^ substituent remained a hydrogen atom, however, a methyl group was tolerated in one example, resulting in two contiguous chiral quaternary carbon centres being formed.

### 4.2. Aziridination

An aziridine is a three-membered nitrogen-containing heterocycle of considerable interest in terms of organic synthesis due to its potential role in performing as a precursor to complex bioactive molecules. Considering ring strain exists within the atoms of the heterocycle, this can provide a way to interesting synthetic pathways which could potentially formulate structurally important pharmaceuticals. The chemistry pertaining to the field of aziridine synthesis has followed many avenues since the first synthesis by Gabriel in 1888 [44,45,46,47,48,49,50,51]. Aziridines are susceptible to ring opening reactions due to the ring strain and the polarized nature of the carbon–nitrogen bond. Aziridines have the capability of partaking in stereoselective ring-opening reactions and cycloadditions, similar to their epoxide analogues, resulting in structurally interesting heterocycles [46,47,48,49,50,51]. Due to the fact that stereoselective aziridination methodologies are sparse and lacking in efficacy, aziridines are less commonly used in synthetic applications compared to their epoxide counterparts [52,53,54]. The utility of sulfonium ylides and sulfoxonium ylides in facilitating asymmetric aziridinations has been described in several reviews in previous years [8,55,56,57,58,59,60,61,62,63]. In analogous fashion to epoxidation, the reaction proceeds through a zwitterionic intermediate formed through nucleophilic addition of the ylidic carbanion to an electrophilic imine site. An intramolecular cyclization occurs, constructing the aziridine.

**Scheme 18:**
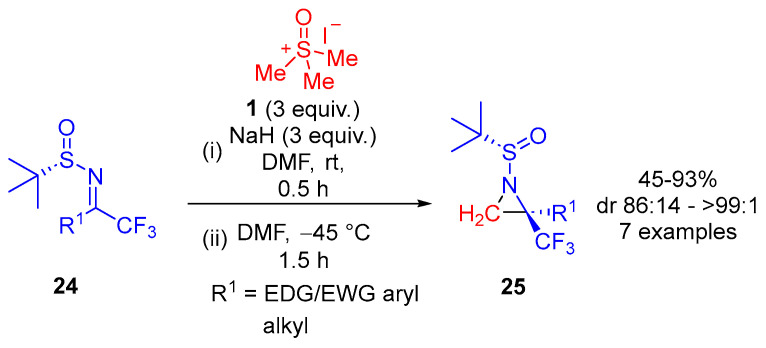
Huang’s asymmetric synthesis of trifluoromethylated aziridines from sulfoxonium ylides and (*S*)-*N*-tert-butanesulfinyl ketimines.

In 2013, Huang and colleagues developed a convenient and practical method for the asymmetric synthesis of trifluoromethylated aziridines by employing reactions of sulfoxonium ylide **2** with (*S*)-*N*-tert-butanesulfinyl ketimines **24** (Scheme 18) [64]. The reaction of ylide generated from trimethylsulfoxonium iodide **1** in the presence of base with chiral tert-butanesulfinyl ketimines **24** furnished trifluoromethylated aziridines **25** in moderate to excellent yields and with very good diastereoselectivities (Table 2). Aromatic as well as aliphatic ketimines underwent the reaction smoothly to afford the corresponding aziridines in good yields (61–93%). In general, electron-withdrawing groups were beneficial to the construction of trifluoromethylated aziridines with good diastereoselectivities but lower yields (45%, 54% and 61%), whereas ketimines bearing an aryl group with an electron-donating group gave the highest yield but with lower diastereoselectivity. The highest diastereoselectivity of de >98% was reached in the cases of the formation of acetylenic trifluoromethylated aziridines, which were afforded in moderate to good yields.

**Scheme 19:**
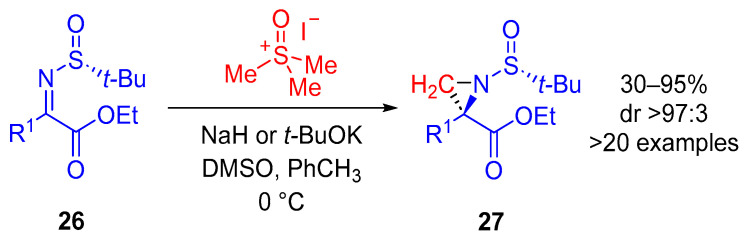
Marsini’s diastereoselective aza-Johnson–Corey–Chaykovsky aziridination.

In 2015, Marsini and colleagues, building on work from Huang, developed a highly diastereoselective aza-Johnson–Corey–Chaykovsky reaction of chiral *N*-tert-butanesulfinyl ketimino esters **26** with dimethylsulfoxonium methylide, to form *α*-quaternary aziridine-2-carboxylates **27** (Scheme 19) [65]. The reaction displayed moderate to excellent yields, exhibiting a broad scope with respect to substrates, and was tolerant of alkyl, alkenyl, aryl and heteroaryl groups substituted at the R^1^ position. The reaction mechanistic pathway is expected to proceed through the conventional route whereby a methylene group undergoes transfer to the imine, forming an aziridine, in <10 min reaction time at 0 °C. They observed, through mechanistic studies, that the reaction proceeds to only form a single diastereomer (*S*,*S*). In terms of which substrates performed most predominantly, the results suggested bulky or aryl-based substituents produced the best yields and diastereoselectivities [65] (Table 3), with smaller substituents showing poorer yields and diastereoselectivities. This methodology provided an avenue to synthesize unnatural heterocyclic amino esters and *α*-quaternary conjugated peptides through follow-up reactions.

**Scheme 20:**
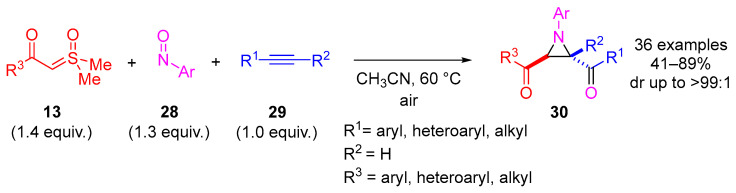
Li’s diastereoselective synthesis of chiral diketo aziridines.

In 2023, Li and colleagues reported a catalyst-free one-pot synthesis of chiral diketo aziridines **30** and alkenes **31** [66]. A three-component reaction of *α*-ketosulfoxonium ylides **13**, nitrosoarenes **28** and terminal alkynes **29** was observed to facilitate access to diketo aziridines **30** with moderate to good *trans*-diastereoselectivities and with moderate to good yields (41–89%) (Scheme 20). They initially investigated the effect of solvent variation in the reaction, and it was clear that CH_3_CN resulted in the best yields (up to 89%) and diastereoselectivity (dr up to >99:1). In contrast, toluene, CH_2_Cl_2_ and THF resulted in yields of 31%, 42% and 50%, respectively, and as a stereorandom mixture of *cis*- and *trans*-diastereomers. Following this, the R^3^ substituent scope of the *α*-ketosulfoxonium ylide was investigated, with the reaction demonstrating tolerance for alkyl, heteroaryl and aryl (with EDG/EWG) groups (Table 4). Activated and deactivated aryl-substituted nitroso substrates were tolerated. However, when 4-nitrosopyridine was investigated under the optimal reaction conditions, no aziridine was formed.

**Scheme 21:**
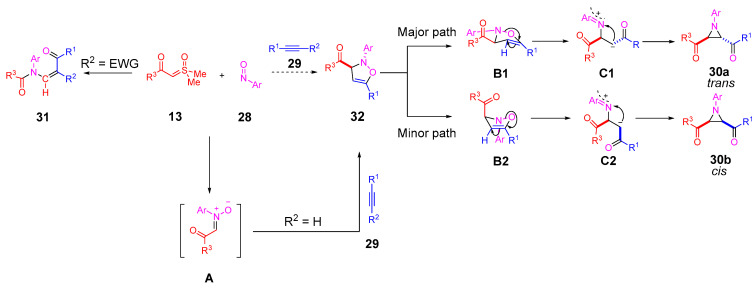
Li’s synthesis of chiral diketo aziridines from nitrosoarenes and terminal alkynes.

The proposed mechanism proceeds via an initial nitrone formation step involving reaction of the *α*-ketosulfoxonium ylide **13** and the nitrosoarene **28** (Scheme 21). The nitrone intermediate **A** reacts with a terminal alkyne **29** in a (3 + 2)-cycloaddition step to form an isoxazoline intermediate **32**. At this point, the reaction pathway can take three possible routes. In the case of terminal alkynes **29** (R^3^ = H) in the reaction, the formation of diketo aziridines has been reported. The isoxazoline intermediate **32** can exist in two different conformations (**B1** and **B2**), and the selectivity for either a *trans*- or *cis*-diastereomer is believed to be due to N-O bond Baldwin rearrangement of the preferred isoxazoline conformation **B1** [66,67]. The preferred *trans*-diastereomer **30a** forms as a result of the two keto carbonyl groups avoiding steric interactions in intermediate **C1**, whereas the formation of the lesser observed *cis*-diastereomer **30b** is due to the slight tolerance of steric repulsion of these groups. In the case of an internal alkyne with an electron-withdrawing group (R^2^) on **29**, the formation of *α*,*β*-substituted ketones **31** was observed. The rationale for this transformation over aziridine formation is due to the stabilizing effect of the electron-withdrawing group during the Baldwin rearrangement step.

### 4.3. Cyclopropanation

**Scheme 22:**
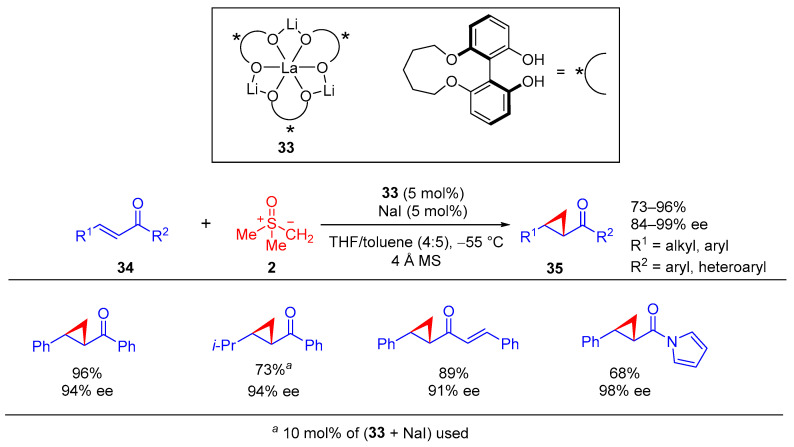
Shibasaki’s catalytic asymmetric synthesis of cyclopropanes.

Shibasaki and colleagues introduced La-Li_3_-(biphenyldiolate)_3_/NaI complex (**33**) as the chiral Lewis acid catalyst for catalytic asymmetric cyclopropanation reaction of enones or *α*,*β*-unsaturated pyrrole amides **34** (Scheme 22) [68]. This methodology furnished diverse functionalized cyclopropane derivatives **35** in high yields and with excellent enantioselectivities. In this methodology, the solution of dimethylsulfoxonium methylide **2** was prepared from trimethylsulfoxonium chloride analogue of **1** by treatment with NaH in THF, followed by filtration (× 2). This procedure helped to avoid undesired salt effects for the Lewis acid-catalyzed asymmetric cyclization reaction.

**Scheme 23:**
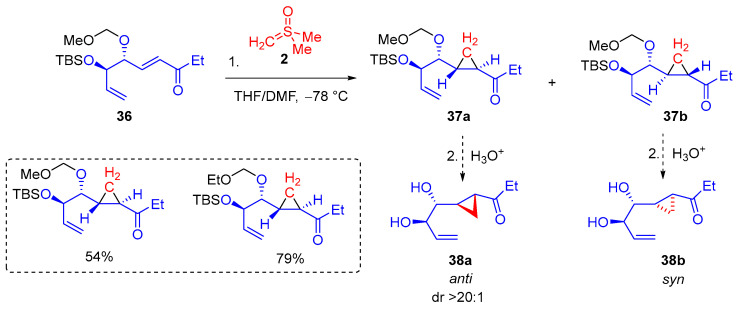
Hou’s diastereoselective synthesis of cyclopropanes leading to the formal synthesis of (−)-brevipolide H.

**Scheme 24:**
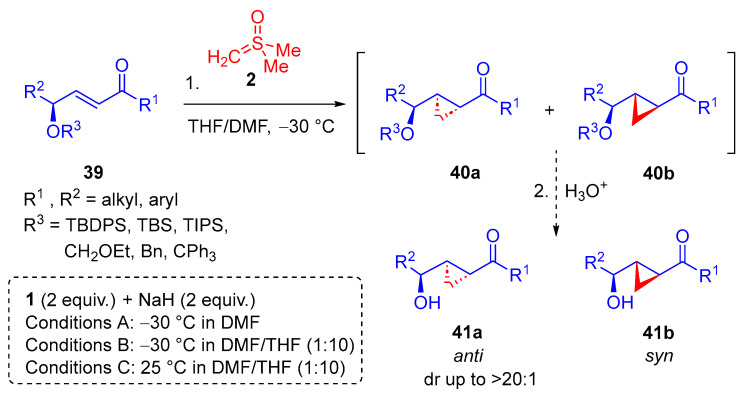
Hou’s diastereoselective synthesis of cyclopropanes leading to the formal synthesis of an eicosanoid.

In 2014, Hou and colleagues reported the diastereoselective synthesis of cyclopropanes through the *anti*-addition of dimethylsulfoxonium methylide **2** to *α*,*β*-unsaturated ketones **36** as part of the first formal synthesis of (−)-brevipolide H **43 [69]**. The reaction was shown to operate most effectively using a solvent system of THF and DMF (20:1) at −78 °C for 6–8 h. After deprotection, **38a** was afforded with high diastereoselectivity (Scheme 23). In addition to the work carried out in 2014, they reported a diastereoselective *anti-*addition of dimethylsulfoxonium methylide **2** to an *α*,*β*-unsaturated ketone **39** in the formal synthesis of an eicosanoid **42 [70]**. A number of enones were probed, and three different sets of reaction conditions were developed to suit substrates, substituted with a particular protecting group (Scheme 24).

**Scheme 25:**
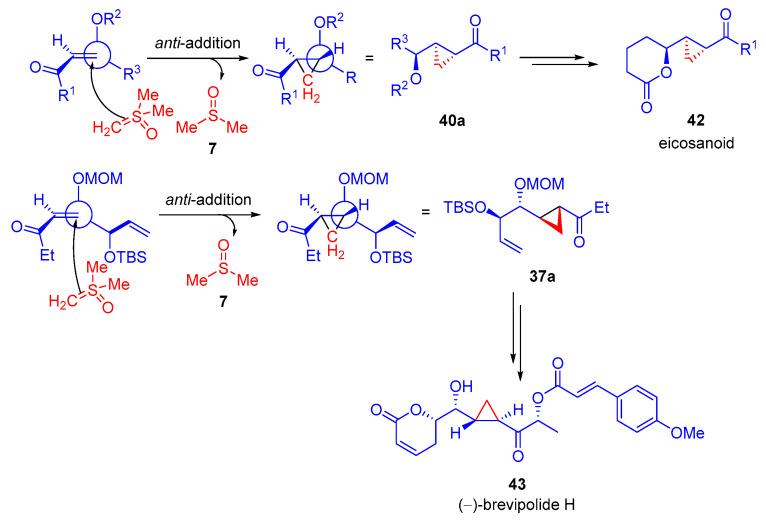
Diastereoselective *anti*-addition of dimethylsulfoxonium ylide to *α*,*β*-unsaturated ketone leading to the synthesis of eicosanoid and (−)-brevipolide H.

The diastereoselectivity observed in the formal synthesis of both (−)-brevipolide H **43** and an eicosanoid **42** is understood to be as a result of *anti-*addition of dimethylsulfoxonium ylide **2** to the *α*,*β*-unsaturated carbonyl starting material (**36** and **39**) (Scheme 25). This occurs due to the bulky alkoxy group being perpendicular to the alkene, which sterically hinders the methylene transfer trajectory occurring in *syn*-addition and promotes *anti*-addition from the less sterically hindered side. The favored spatial arrangement of the alkoxy group to the alkene maximizes the overlap of the C-O *σ*-bond and alkene π*-orbital.

**Scheme 26:**
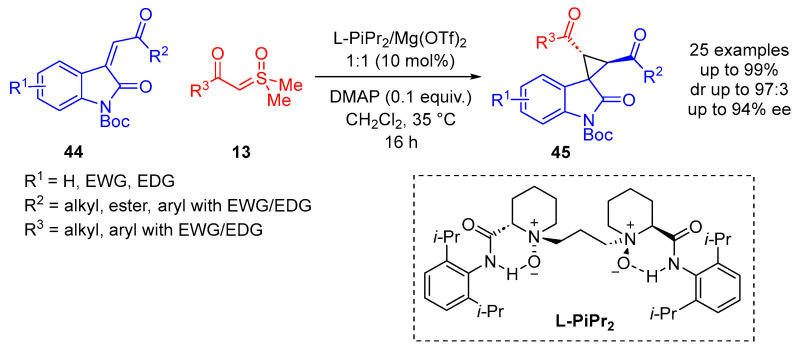
Feng’s catalytic asymmetric synthesis of spiro-cyclopropyl oxindoles.

In 2018, Feng and colleagues reported the catalytic asymmetric synthesis of chiral spiro-cyclopropyl oxindoles **45** in good yields of up to 99%, with good diastereoselectivity and high enantioselectivity (Scheme 26) [71]. This methodology uses a chiral *N*,*N*-dioxide/Mg(OTf)_2_ complex. Nickel(II) and zinc(II) catalysts were probed when the reaction was initially optimized, both of which provided moderate yields (66% and 69%, respectively) and good diastereoselectivity (dr 93:7 and dr 95:5, respectively). L-PiPr_2_ was determined to be the best chiral ligand when utilized with Mg(OTf)_2_ in CH_2_Cl_2_ at 35 °C for 16 h, providing a yield of 99%, diastereoselectivity of 92:8 and enantioselectivity of 91% ee. They reported 25 substrate examples, exploring the scope of the sulfoxonium ylides **13** and 3-phenacylideneoxindoles **44** [71]. Both electron-donating and electron-withdrawing groups were tolerated when substituted on the oxindole backbone (R^1^). Alkyl, ester and aryls bearing electron-donating or electron-withdrawing groups were tolerated when probed as the R^2^ and R^3^ substituents.

**Scheme 27:**
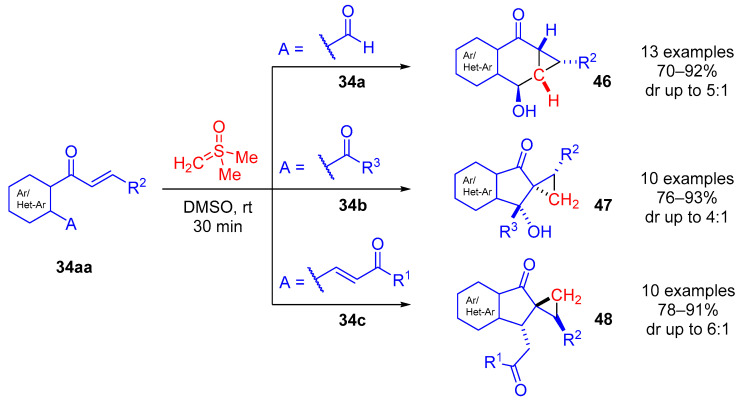
Ramasastry’s diastereoselective synthesis of cyclopropane-fused tetralones and indeno spirocyclopropanes from interrupted Corey–Chaykovsky reactions.

As discussed in the Epoxidation section (Section 4.1), the developing area of interrupted Johnson–Corey–Chaykovsky reactions has seen success in the context of cyclopropanation reactions [40,41,42]. In 2019, Ramasastry and colleagues developed an interrupted Johnson–Corey–Chaykovsky reaction methodology in which they discovered the unexpected formation of cyclopropanoids through the reaction of dimethylsulfoxonium methylide **2** (1.2 equiv.) with *α*,*β*-unsaturated aryl ketones **34aa** in DMSO for 30 min at room temperature (Scheme 27) [40]. It was reported that, depending upon the functional group in the *ortho*-position, an unexpected cyclopropanoid product would be formed. Three different *α*,*β*-unsaturated aryl ketones were probed in reaction with a sulfoxonium ylide. Cyclopropane-fused tetralones **46** were observed to form in the case where a formyl group was placed in the *ortho*-position of *α*,*β*-unsaturated ketone **34**, contrary to the expected cyclopropanation of the *α*,*β*-unsaturated ketone, or indeed, epoxidation of the carbonyl group.

**Scheme 28:**
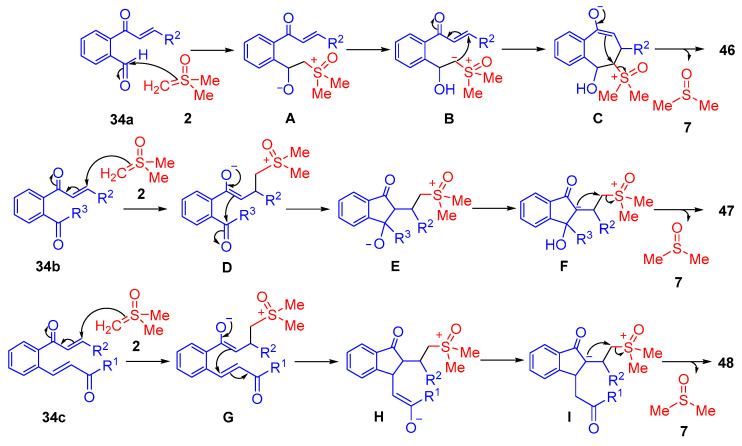
Proposed mechanisms for Ramasastry’s interrupted Johnson–Corey–Chaykovsky reactions.

The plausible reaction mechanism starts with an initial 1,2-addition of dimethylsulfoxonium methylide **2** to the carbonyl group of the aldehyde **34a**, which is followed by a 1,3-H^+^ transfer in **A** (Scheme 28). The ylidic carbanion of intermediate **B** carries out a 1,4-addition in intramolecular fashion. The resulting enolate of intermediate **C** nucleophilically attacks the carbon adjacent to the sulfoxonium group, which forms a fused cyclopropane ring **46**, with concomitant loss of DMSO **7**. Overall, 13 examples were reported with good to excellent yields (70-92%) and good diastereoselectivity (dr up to 5:1). When a ketone group was substituted in the *ortho-*position in **34b**, an indene-type spirocyclopropane **47** was observed to form. The proposed reaction mechanism starts with a 1,4-addition by sulfoxonium ylide **2**, and the resulting enolate of intermediate **D** carries out a 1,2-addition to the carbonyl of the ketone. A 1,5-H^+^ transfer of **E** occurs to afford **F**, followed by the nucleophilic addition of the carbanion to the carbon adjacent to the sulfoxonium group of **F**, resulting in a spiro-cyclopropane **47** (Scheme 28). In total, 10 examples were reported with good to excellent yields (76–93%) and moderate diastereoselectivity (up to dr 4:1). In the case of unsymmetrical dienones **34c**, the reaction proceeds similarly through an initial 1,4-addition by dimethylsulfoxonium methylide **2**. An intramolecular cyclization occurs through a second 1,4-addition by the enolate of intermediate **G**. Following this, a 1,5-H^+^ transfer of **H** occurs to afford **I**, which allows the resulting carbanion to nucleophilically attack the carbon adjacent to the sulfoxonium group of **I**, leading to the formation of an indene-type spirocyclopropane **48** (Scheme 28). Overall, 10 examples were reported with good to excellent yields (78–91%) and moderate to good diastereoselectivity (dr up to 6:1). The three reactions demonstrated tolerance for alkyl, heteroaryl and aryls bearing EWG/EDG groups substituted in the R^1^, R^2^ and R^3^ positions. The aryl backbone also showed tolerance for EDG/EWG-substituted aryls and heteroaryls.

**Scheme 29:**
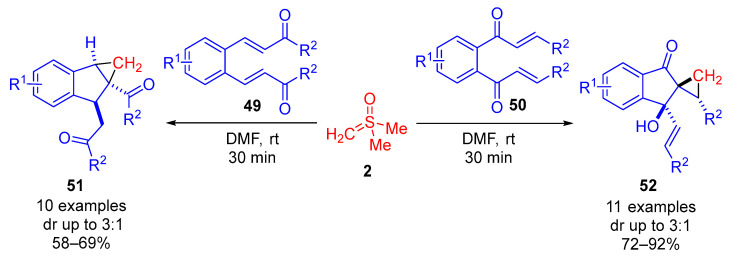
Ramasastry’s interrupted Johnson–Corey–Chaykovsky reaction of symmetrical dienones.

In 2019, Ramasastry and colleagues further explored the reaction between dimethylsulfoxonium methylide **2** and symmetrical aryl dienones (Scheme 29) [41]. The paper described the unexpected synthesis of cyclopropanoids through desymmetrization by sulfoxonium ylides. The reaction proceeded optimally with 1.2 equivalents of sulfoxonium ylide in DMF at room temperature for 30 min. When the symmetrical dienone with terminal ketone groups **49** was treated with sulfoxonium ylide **2** under these conditions, 10 examples of cyclopropane-fuse indanes **51** were formed in good reaction yields (58−69%) and with moderate diastereoselectivity (dr up to 3:1). Symmetrical dienones with terminal vinyl groups **50** produced 11 examples of indeno-spirocyclopropane **52** in good to excellent reaction yields (72–92%) and with moderate diastereoselectivity (dr up to 3:1). The paper reported substrate tolerance for halogen-, alkyl- and methoxy-substituted aryls in the R^1^ and R^2^ positions.

**Scheme 30:**
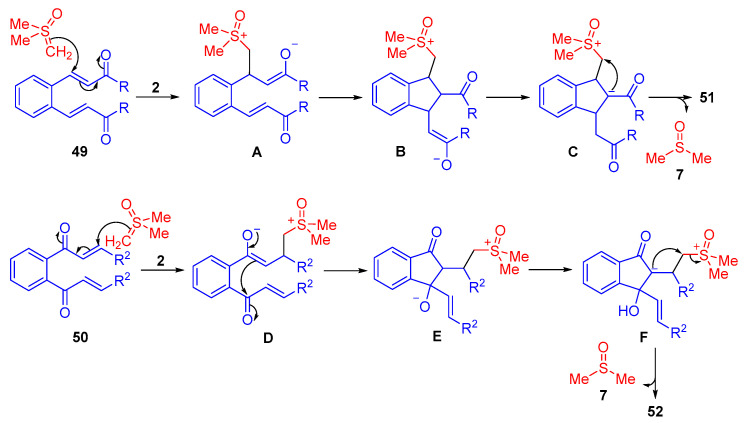
Proposed mechanisms of Ramasastry’s interrupted Johnson–Corey–Chaykovsky reactions of symmetrical dienones.

The reaction mechanism for the unexpected synthesis of cyclopropane-fused indanes from symmetrical dienones with terminal ketone groups **49** proceeds with an initial 1,4-addition by the sulfoxonium ylide **2** (Scheme 30). The enolate of intermediate **A** intramolecularly cyclizes through another 1,4-addition. A 1,3-H^+^ transfer of **B** occurs, which allows the resulting carbanion of **C** to displace DMSO **7** and form a cyclopropane-fused indane **51** (Scheme 30). In the case of terminal vinyl groups **50**, the reaction also starts through an initial 1,4-addition by a sulfoxonium ylide **2**. A 1,2-addition occurs between the enolate of the resulting intermediate **D** to the opposite carbonyl group of the ketone. Following this, a 1,3-H^+^ transfer of **E** occurs and allows for the displacement of DMSO **7** by the carbanion of **F** to form an indene-type spirocyclopropane **52** (Scheme 30).

**Scheme 31:**
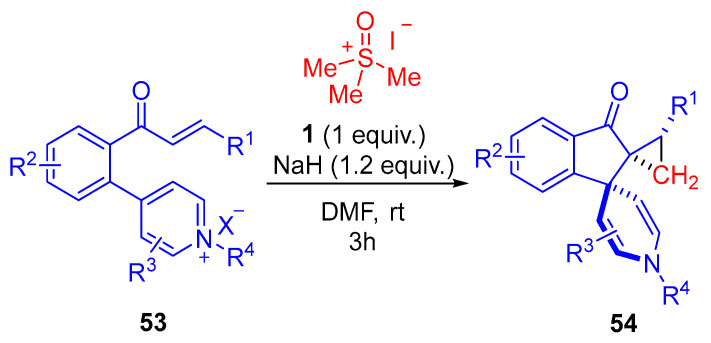
Ramasastry’s unexpected synthesis of vicinal bis-spirocyclic indanones.

In 2023, Ramasastry and colleagues further explored the interrupted Johnson–Corey–Chaykovsky reactions of sulfoxonium ylides **2** with *α*,*β*-unsaturated aryl ketones bearing a pyridinium salt in the *ortho*-position of **53** (Scheme 31) [42]. The reaction functioned most optimally in DMF at room temperature for 3 h using 1 equivalent of trimethylsulfoxonium iodide **1** and 1.2 equivalents of NaH to form the sulfoxonium ylide **2**. When the starting substrate **53** was placed under these conditions, an unexpected synthesis of vicinal bis-spirocyclic indanones **54**, providing three contiguous quaternary carbons, was obtained. The group reported 23 examples of vicinal bis-spirocyclic indanones with moderate to good reaction yields (49–81%) and good diastereoselectivity (dr up to 9:1). The reaction demonstrated substrate tolerance for EDG/EWG substituted aryls and heteroaryls in the R^1^ and R^2^ positions, alkyl groups in the R^3^ position, as well as alkyl groups, EDG-substituted aryls and heteroaryls in the R^4^ position.

**Scheme 32:**
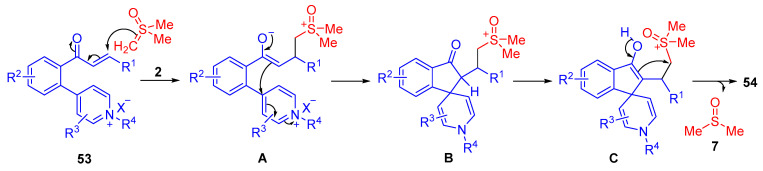
Proposed mechanism for Ramasastry’s interrupted Johnson–Corey–Chaykovsky reactions of sulfoxonium ylides and pyridinium salts.

The proposed reaction mechanism starts with an initial 1,4-addition by dimethylsulfoxonium methylide **2**, resulting in intermediate **A** (Scheme 32). The enolate of **A** derived from **53** intramolecularly cyclizes by nucleophilically attacking C4 of the pyridinium ring, which forms an indanone ring. The keto carbonyl group of **B** tautomerizes to form an enol **C**. The enol nucleophilically attacks the carbon adjacent to the sulfoxonium group, allowing the leaving group to cleave and form the desired vicinal bis-spirocyclic indanone **54**.

**Scheme 33:**
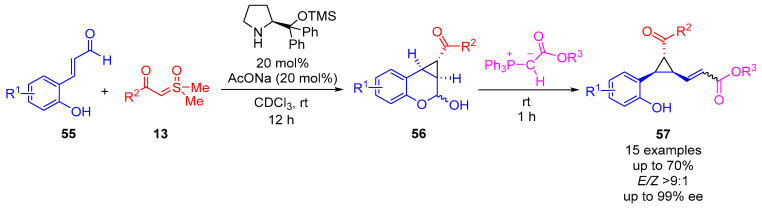
Bernardi’s asymmetric aminocatalyzed synthesis of cyclopropane-fused chromanol.

In 2022, Bernardi and colleagues developed a methodology for the enantioselective synthesis of chiral-fused cyclopropane-chromane scaffolds **56**, named 1,1a,2,7b-tetrahydrocyclopropa[c]chromenes, through aminocatalysis using sulfoxonium ylides **13** with 2-hydroxycinnamaldehydes **55** (Scheme 33) [72]. This method utilized the Jørgensen–Hayashi catalyst in a catalytic amount (20 mol%), along with NaOAc (20 mol%), in deuterated chloroform at room temperature for 12 h. NaOAc, as an additive, was found to facilitate the best combination of enantioselectivity and reaction yield. Benzoic acid and acetic acid were also trialed and performed well, giving good reaction yields and strong enantioselectivity. The cyclopropane ring was formed with perfect diastereoselectivity. The fused cyclopropane–chromane intermediate **56**, formed through aminocatalysis, underwent a Wittig olefination at room temperature for 1 h to produce 1,2,3-trisubstituted cyclopropanes **57**, exhibiting 15 examples with moderate to good yields (up to 70%), excellent enantioselectivity (up to 99% ee) and good *E*-selectivity (*E/Z* > 9:1). The reaction showed tolerance for alkyl and activating groups substituted in the *meta-* and *para*- positions, in addition to the *ortho*- hydroxyl group, for R^1^. Ester groups and ketone groups were investigated in the R^2^ position for the ylide **13**, with *α*-estersulfoxonium ylides observed to perform better than *α*-ketosulfoxonium ylides.

**Scheme 34:**
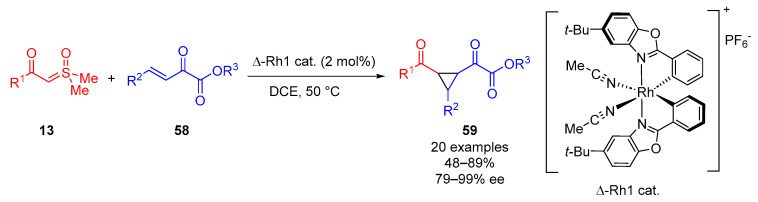
Li’s asymmetric catalytic synthesis of 1,2,3-trisubstituted cyclopropanes.

In 2022, Li and colleagues carried out the asymmetric synthesis of cyclopropanes from *α*-ketosulfoxonium ylides **13** and *β*,*γ*-unsaturated ketoesters **58** using a chiral Rh(III) catalyst (Scheme 34) [73]. The synthesis of 20 examples of chiral 1,2,3-trisubstituted cyclopropanes **59** was reported to proceed with moderate to excellent yields (48–89%), high enantioselectivity (79–99% ee) and excellent diastereoselectivity (dr > 20:1). Following optimization studies, the most effective rhodium catalyst (Δ-Rh1 cat.) facilitated the best yield, diastereoselectivity and enantioselectivity when the reaction was carried out in 1,2-dichloroethane (75%, dr > 20:1, 97% ee) at 50 °C for 45 h under an argon atmosphere. Toluene facilitated high diastereoselectivity (dr > 20:1) and high enantioselectivity (91% ee), albeit with a moderate reaction yield of 58%. THF facilitated high diastereoselectivity (dr > 20:1) but moderate enantioselectivity (40% ee), and a poor product yield of 10% was obtained. Alkyl, electron-donating/electron-withdrawing group-substituted aryl and heteroaryl groups were tolerated when probed as the R^1^ and R^2^ substituents.

**Scheme 35:**
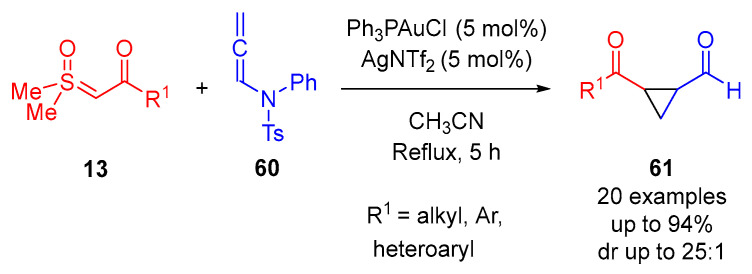
Zhao’s diastereoselective cyclopropanation of allenamides with sulfoxonium ylides through Au-catalysis.

In 2023, Zhao and colleagues reported the synthesis of cyclopropanes **61** through an Au-catalyzed (2 + 1)-cycloaddition of allene-type tosylamide **60** with sulfoxonium ylides **13** (Scheme 35) [74]. A series of *α*-ketosulfoxonium ylides were probed in reaction with tosylamide **60**, resulting in 20 examples with reaction yields of up to 94% and good diastereoselectivity (up to dr 25:1). The reaction was initially optimized to determine the most suitable Au-catalyst for the synthesis of 1-benzoyl-2-formylcyclopropane. A mixture of PhPAuCl (5 mol%) and AgNTf_2_ (5 mol%) in CH_3_CN provided the best results when refluxed for 5 h, whereas using Ph_3_PAuCl (5 mol%) or AgNTf_2_ (5 mol%) singularly resulted in no reaction. CH_3_CN was observed to afford the best reaction yield, with toluene, CH_2_Cl_2_, THF and EtOAc providing moderate yields (38–58%). The substrate scope was investigated through the exploration of 20 different *α*-ketosulfoxonium ylides **13**, showing tolerance for the alkyl, heteroaryl and aryl groups (as R^1^). Both electron-donating and electron-withdrawing group-substituted aryls were tolerated.

**Scheme 36:**
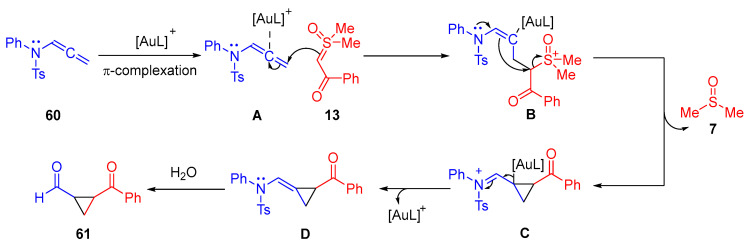
Proposed reaction mechanism of Zhao’s diastereoselective cyclopropanation.

The plausible reaction mechanism starts with an initial *π*-complexation of the cationic gold-catalyst to the allene **60** (Scheme 36). Following this, *α*-ketosulfoxonium ylide **13** nucleophilically attacks the terminal end of the Au-complexed allene **A**. The resulting Au-substituted enamide intermediate moiety intramolecularly attacks the carbon adjacent to the sulfoxonium group in **B**, eliminating DMSO **7** and forming **C**. An enamide **D** forms as a result of the cationic Au-catalyst being eliminated. Intermediate **D** then undergoes acid-catalyzed hydrolysis to form the formylated cyclopropane product **61**.

**Scheme 37:**
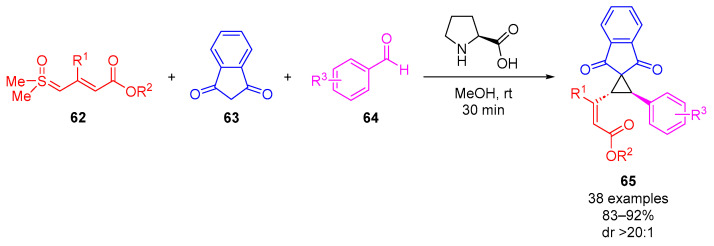
Vaitla’s diastereoselective cyclopropanation of aryl aldehydes, indane-1,3-dinones and vinyl sulfoxonium ylides using aminocatalysis.

Vaitla and colleagues developed the L-proline-catalyzed diastereoselective reaction sequence involving cyclopropanation using aryl aldehydes **64**, indane-1,3-diones **63** and vinyl sulfoxonium ylides **62** (Scheme 37) [75]. They reported 38 examples of 1,2,3-trisubstituted cyclopropanes **65** obtained in excellent yields (83–92%) and with excellent diastereoselectivity (dr > 20:1). The cyclopropanation reaction functioned most effectively when carried out in MeOH at room temperature for 30 min using L-proline as catalyst (20 mol%), 1.4 equivalents of sulfoxonium ylide **62**, 1.1 equivalents of aldehyde **64** and 1 equivalent of indane-1,3-dione **63**. A series of different catalysts were trialed in place of L-proline, and all showed excellent diastereoselectivity (dr > 20:1). Indium(III) triflate, scandium(III) triflate and triethylamine resulted in poor reaction yields of 15%, 22% and 34%, respectively. Tetrafluoroboric acid diethyl ether complex performed effectively to give a reaction yield of 85%. DMSO, CH_3_CN and EtOAc were explored as solvents and gave good yields of 80%, 65% and 78%, respectively. In terms of substrate scope, the reaction was tolerant of EDG/EWG-substituted aryl, alkyl and heteroaryl aldehydes as well as EDG/EWG-substituted aryl at the *β*-position (R^1^) of vinyl sulfoxonium ylides.

### 4.4. Olefination

The C=C double bond is a central functional group in organic chemistry due to its importance and application in total synthesis [76]. Typical methodologies to construct C=C double bond include the Wittig reaction, Peterson, Julia, Tebbe olefinations and the Horner–Wadsworth–Emmons reaction by employing phosphonium ylides/phosphonate anions, silicon-stabilized carbanions, sulfones or metal alkylidenes as reagents [77,78,79,80,81]. All these reactions proceed through cyclic intermediates, either a four-membered ring or five-membered spirocyclic intermediate.

**Scheme 38:**
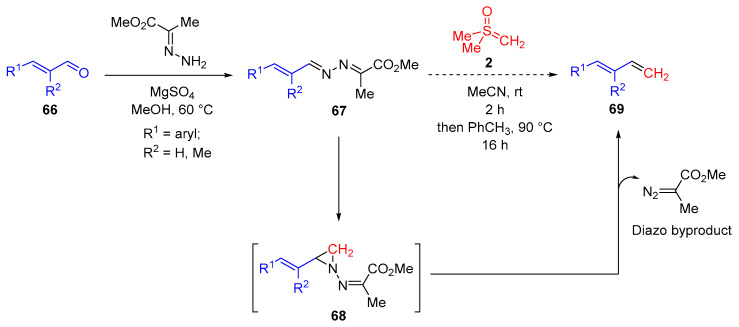
Synthesis of alkenes using sulfoxonium ylides.

Very recently, Maulide and colleagues reported a three-membered ring approach to carbonyl olefination (Scheme 38) [82]. They applied a one-pot procedure to access the key intermediate, a three-membered *N*-iminyl aziridine **68**, by addition of a sulfoxonium ylide **2** to an in situ-generated *N*-iminyl imine (azine) **67** from the corresponding aldehyde **66**. Cheletropic cycloreversion or thermal decomposition of the N-iminyl aziridine **68** led to the desired alkene **69** in good to high yields. This methodology tolerated substrates ranging from aromatic aldehydes, aliphatic aldehydes and *α*,*β*-unsaturated aldehydes to sensitive *α*-chiral aldehydes. The *E-*isomer selectivity of the alkene products might be explained by involvement of the more stable *trans*-aziridine intermediate or non-concerted ring-opening pathways causing isomerization. The procedure was not successful for trisubstituted olefin synthesis because the required aziridine could not be obtained from either a branched sulfoxonium salt or from a ketone. However, the reaction provides a convenient alternative to Wittig-type methylenation in that it avoids the generation of difficult-to-separate triphenylphosphine oxide byproduct.

**Scheme 39:**
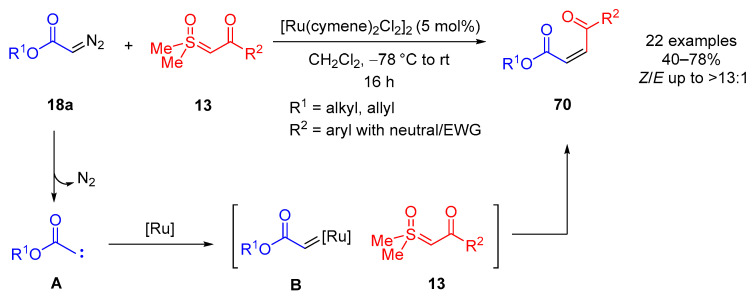
Maulide’s ruthenium-catalyzed cross-olefination of sulfoxonium ylides and *α*-diazoesters.

In addition to this, they reported a methodology for the stereoselective synthesis of 1,2-disubstituted alkenes **70** from *α*-diazoesters **18a** and *α*-ketosulfoxonium ylides **13** through ruthenium catalysis (Scheme 39) [83]. It was reported that 22 examples were obtained in moderate to good reaction yields (40–78%), and very good *Z*-selectivity (*Z/E* up to >13:1) was also demonstrated (Table 5). The reaction showed tolerance for bulky alkyl ester groups substituted at the R^1^ position as well as neutral and EWG-substituted aryls at the R^2^ position. The stereoselective cross-olefination proceeds through initial formation of a carbene **A**, generated from the loss of nitrogen of an *α*-diazoester **18a**. A metal carbenoid **B** was formed through the combination of the carbene and the [Ru(cymene)_2_Cl_2_]_2_ catalyst. The metal carbenoid coupled with the sulfoxonium ylide **13**, allowing the formation of the *Z*-olefin product **70**.

**Scheme 40:**
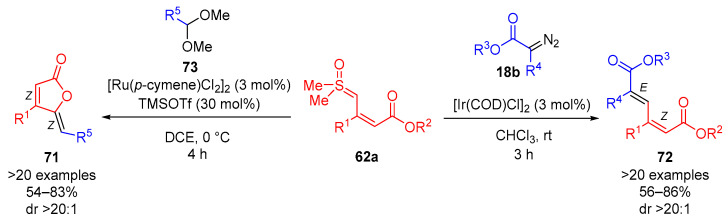
Vaitla’s stereoselective olefination of vinyl sulfoxonium ylides with diazo compounds and acetals.

In 2024, Vaitla and colleagues reported a diastereoselective synthesis of conjugated dienoates **72** from *α*-diazoesters **18b** and vinyl sulfoxonium ylides **62a** using iridium-catalysis (Scheme 40) [84]. In addition, the paper described a diastereoselective synthesis of ylidene butenolides **71** from acetals **73** and vinyl sulfoxonium ylides **62a** using ruthenium-catalysis aided by TMSOTf. The synthesis of conjugated dienoates **72** provided >20 examples with excellent diastereoselectivity (dr > 20:1) and in moderate to very good reaction yields (56–86%). The reaction was observed to function most efficiently in CHCl_3_ at room temperature for 5 h, using equimolar amount of sulfoxonium ylide **62a** and *α*-diazoester **18b** and 3.0 mol% of [Ir(COD)Cl]_2_. The conjugated dienoates **72** obtained were formed as the *Z*,*E*-isomer, which was congruent with *cis*,*trans*-muconic acid. The reaction was tolerant of EDG-substituted and EWG-substituted (R^1^, R^5^) aryls. This work reported >20 examples of ylidene butanolides **71** formed with excellent diastereoselectivity (dr > 20:1) and in favorable yields (54–83%). The optimal reaction conditions were determined to be DCE as solvent at 0 °C for 4 h, using 3.0 mol% of [Ru(*p*-cymene)Cl_2_]_2_ and 30 mol% of TMSOTf. The diastereoselectivity of the ylidene butenolide **71** synthesis favored the formation of the *Z*,*Z-*isomer, which is congruent with the structure of rubrolide analogues. This reaction was observed to be tolerant of heteroaryls and EWG- and EDG-substituted (R^1^, R^4^) aryls.

**Scheme 41:**
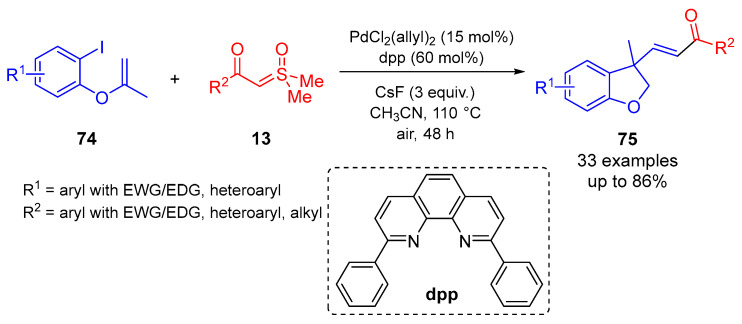
Jiang’s stereoselective olefination through reaction of *β*-ketosulfoxonium ylides and 1-iodo-2-((methylallyl)oxy)benzenes using palladium catalysis.

**Scheme 42:**
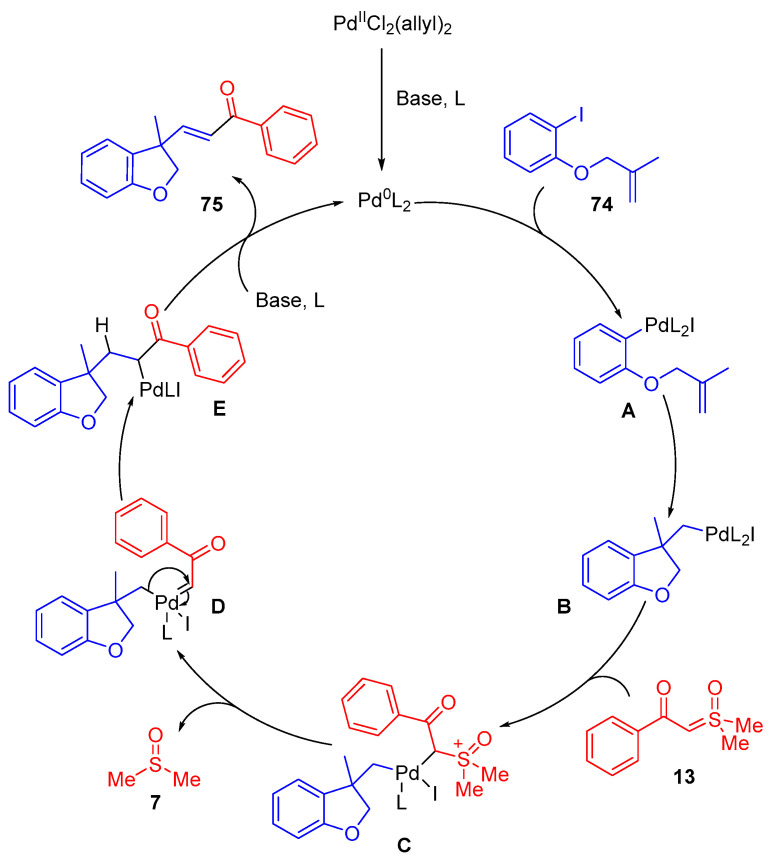
Proposed catalytic cycle of Jiang’s stereoselective olefination.

Jiang and colleagues reported a Pd-catalyzed stereoselective synthesis of *α*,*β*-unsaturated carbonyls from *β*-ketosulfoxonium ylides and 1-iodo-2-((methylallyl)oxy)benzene compounds **74**, which proved to be favorable across a range of substrates for both starting materials (Scheme 41 and Scheme 42, Table 6) [85]. The initial reaction optimization study found that the cross-coupling reaction between *β*-ketosulfoxonium ylides and 1-iodo-2-((methylallyl)oxy)benzene compounds performed best at 110 °C for 48 h in CH_3_CN with the PdCl_2_(allyl)_2_ catalyst (15 mol%), 2,9-diphenyl-1,10-phenathroline ligand (60 mol%) and 3.0 equiv. of CsF, resulting in an 86% yield of benzofuran-type product **75**. Other Pd-based catalysts were probed, including PdCl_2_ and PdBr_2_, and both resulted in lower yields of 18% and 10%, respectively. Several phosphine-based and chelating ligands were examined, with 2,9-diphenyl-1,10-phenathroline performing most effectively. CH_3_CN was determined to produce the best performance when compared to the other solvents investigated, such as DCE, THF and acetone. The reaction was found to perform effectively under an air atmosphere, with no beneficial effect when carried out under a N_2_ or O_2_ atmosphere. A number of Cs bases were examined, with 3.0 equiv. of CsF providing the best results. This work provides an effective stereoselective olefination methodology which favors the formation of *E*-isomers, with moderate to good yields (35–86%) and is tolerant of a wide scope of *β*-ketosulfoxonium ylide **13** and 1-iodo-2-((methylallyl)oxy)benzene **74** starting substrates.

The proposed mechanism proceeds via an initial reduction of the Pd(II)-catalyst using basic CsF and with 2,9-diphenyl-1,10-phenathroline and triphenylphosphine acting as ligands. The Pd(0)-catalyst inserts into the C–I bond of 1-iodo-2-((methylallyl)oxy)benzene **74** in an oxidative addition step. The Pd(II)-complex **A** undergoes intramolecular cyclization to form the 2,3-dihydrobenzofuranyl ring **B**. *β*-ketosulfoxonium ylide **13** associates to the Pd(II)-complex, DMSO **7** is eliminated, and a Pd-carbene **D** is formed. Following a 1,2-alkyl migration, an alkylpalladium complex **E** is formed, which undergoes *β*-hydride elimination to form the *α*,*β*-unsaturated carbonyl product **75** with reforming Pd(0)-catalyst.

**Scheme 43:**
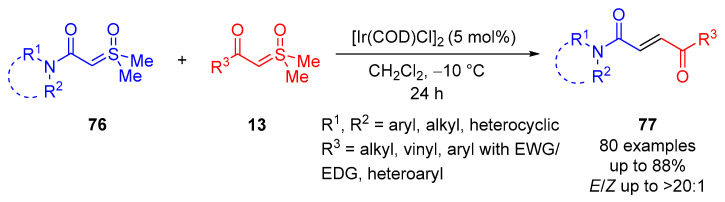
Liao’s diastereoselective synthesis of disubstituted alkenes using iridium-catalysis.

**Scheme 44:**
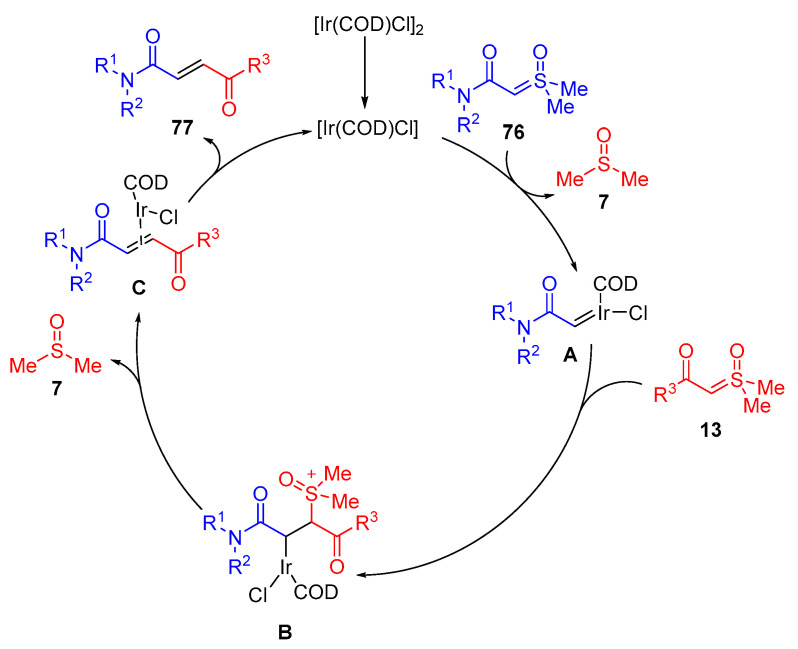
Reaction mechanism of Liao’s iridium-catalyzed cross-dimerization.

Liao and colleagues reported an iridium-catalyzed olefination of sulfoxonium ylides **13**, providing 80 examples with excellent *E*-isomer diastereoselectivity (*E/Z* up to >20:1) of disubstituted alkenes **77** in favorable yields of up to 88% (Scheme 43) [86]. They reported the reactions of *α*-ketosulfoxonium ylides or *α*-estersulfoxonium ylides with *α*-amidesulfoxonium ylides or *N*-heterocyclic *α*-amidesulfoxonium ylides. The most optimal conditions employed were *α*-amidosulfoxonium ylide **76** (1 equiv.) and *α*-carbonylsulfoxonium ylide **13** (1.5 equiv.) in CH_2_Cl_2_ at −10 °C for 24 h under an N_2_ atmosphere. The catalyst employed was [Ir(COD)Cl]_2_ (5 mol%). The proposed reaction mechanism starts with the initial formation of an Ir−carbene intermediate **A** through the reaction of Ir(I) catalyst with ylide **76**, which eliminates DMSO **7** (Scheme 44). An *α*-amidosulfoxonium ylide was observed to form a carbene metalloid faster than an *α*-carbonylsulfoxonium ylide. Following this, an *α*-carbonylsulfoxonium ylide **13** adds to the carbon adjacent to the Ir catalyst to afford **B**. Elimination of DMSO **7** and the Ir catalyst provides **C,** and following catalyst dissociation, *E*-alkene **77** is obtained (Scheme 44). In terms of substrate scope, the reaction was shown to be tolerant of heterocyclic, aryl and alkyl groups in the R^1^ and R^2^ positions. EDG/EWG-substituted aryl, alkyl, vinyl and heteroaryl groups were tolerated in the R^3^ position (Scheme 44, Table 7).

**Scheme 45:**
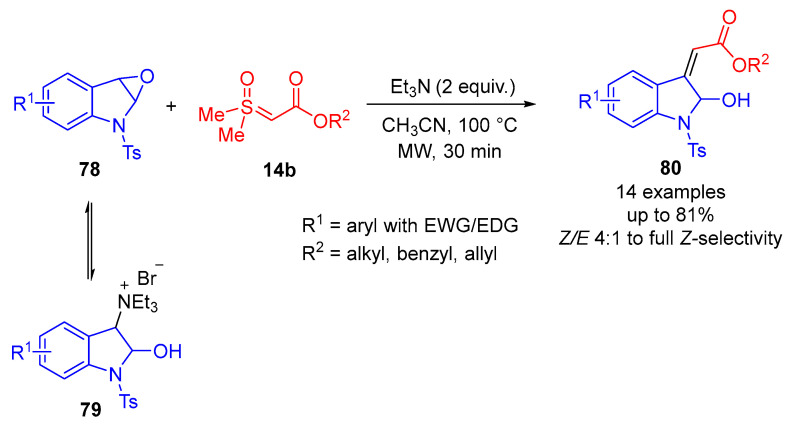
Burtoloso’s diastereoselective synthesis of 2-hydroxyindolin-3-ylidenes **80** from in situ generated epoxide-fused indolines and sulfoxonium ylides.

Recently, in 2024, Burtoloso and colleagues reported a selective ring-opening of epoxide-fused indolines **78** by *α*-estersulfoxonium ylides **14b** (Scheme 45) [87]. Epoxide-fused indolines were unstable and were prepared in situ from 2-hydroxyindoline-3-triethylammonium bromide **79** solution as developed by Abe and colleagues [88]. They detected the formation of an epoxide-fused indoline **78** in solution through NMR studies [87]. The reaction functioned most effectively using two equivalents of triethylamine in CH_3_CN at 100 °C for 30 min under microwave irradiation. When one equivalent of 2-hydroxyindoline-3-triethylammonium bromide **79** and two equivalents of sulfoxonium ylide **14b** were treated under these reaction conditions, 14 olefin examples (**80**) were formed in favorable yields of up to 81% and with moderate to excellent *Z*-selectivity (*Z/E* 4:1 to exclusively *Z*-isomer formation). The reaction demonstrated tolerance for the activated and deactivated indolines (R^1^), as well as the alkyl, benzyl and allyl groups substituted at the R^2^ position.

### 4.5. Insertion Reactions

The most commonly observed mechanistic pathway of sulfoxonium ylides is the formal cycloaddition: (2 + 1) (e.g., epoxides, cyclopropanes, aziridines), (3 + 1) (e.g., oxetanes, azetidines), (4 + 1) (e.g., indanones, indolines). This pathway proceeds through the formation of a betaine intermediate occurring from the nucleophilic addition of the carbanion to an electrophilic site. A cyclic product forms as a result of an intramolecular cyclization. Insertion reactions of sulfoxonium ylides have also been observed to proceed with great success in recent years. This typically occurs as a result of protonation of the sulfoxonium ylide carbanion by X–H bonds (e.g., X = S, N or P), which is followed by a nucleophilic addition of X, to form an inserted product.

**Scheme 46:**
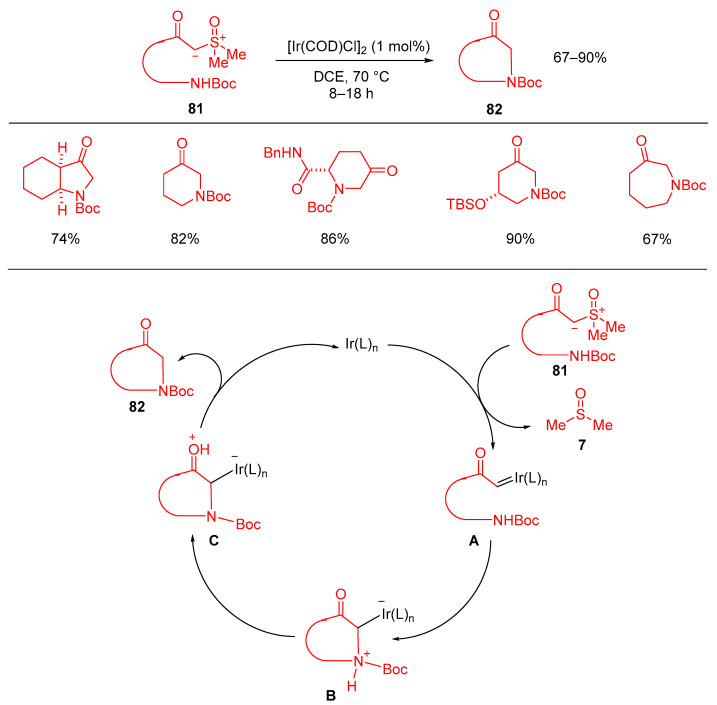
Mangion’s iridium-catalyzed synthesis of aza-heterocyclic products.

Mangion and colleagues reported that transition metal complexes, such as RuCl_2_(Cp)(PPh_3_)_2_, [Ir(COD)_2_Cl]_2_, Pt(COD)Cl_2_ and AuCl(SMe_2_), could be used to produce the corresponding metal carbenoids from sulfoxonium ylides at room temperature (Scheme 46) [89,90]. These metal carbenoids underwent facile reaction with anilines, Boc- or Cbz-protected aliphatic amines, thiophenols and aliphatic alcohols with good efficiency. This method has applications in both intra- and intermolecular reactions, including a practical ring-expansion strategy for lactams. The intramolecular N–H insertion reactions of the carbamate-containing sulfoxonium ylide **81** were developed to facilitate access to structurally complex aza-heterocyclic products **82** [89]. The authors also proposed a possible reaction pathway to explain these transformations: catalyst [Ir(COD)Cl]_2_ reacts with sulfoxonium ylide **81** to generate the metal carbenoid intermediate **A**, and then a subsequent intramolecular N–H insertion generates zwitterionic intermediates **B** and **C**, which, on autoprotolysis, yields the final aza-heterocyclic product **82**.

**Scheme 47:**
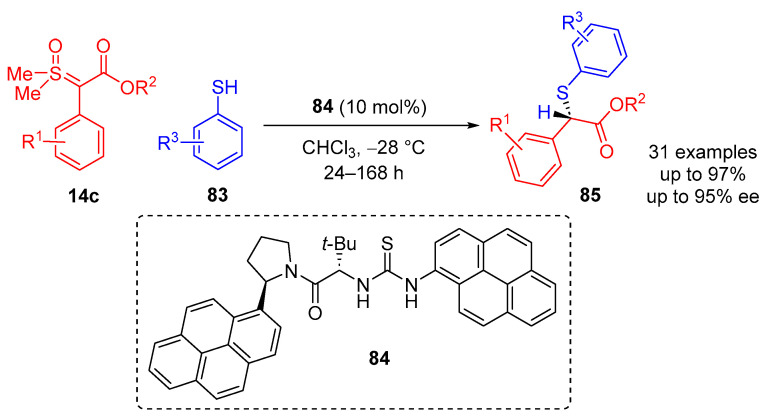
Burtoloso’s enantioselective organocatalytic insertion of *α*-carbonylsulfoxonium ylides and aryl thiols.

In 2020, Burtoloso and colleagues reported an enantioselective organocatalytic insertion of *α*-aryl-*α*-carbonylsulfoxonium ylides **14c** and aryl thiols **83** (Scheme 47) [91]. Due to the acidic nature of thiols, aryl thiols donate a proton to sulfoxonium ylides and the nucleophilic thiolate attacks the carbon adjacent to the sulfoxonium group, eliminating DMSO and resulting in an inserted product **85**. The reported methodology utilizes a chiral thiourea organocatalyst **84** to induce enantiocontrolled addition and is proposed to function using hydrogen-bonding intermolecular interactions to the sulfoxonium ylide. The enantioselectivity of the reaction was explained by a *re*-face protonation of the sulfoxonium ylide. The S–H insertion was discovered to perform most efficiently in CHCl_3_ at −28 °C for 24–168 h, depending on the substrate, using a bulky thiourea **84**. They reported 31 examples of S–H insertions with excellent enantioselectivity (up to 95% ee) and moderate to excellent reaction yields (up to 97%), demonstrating tolerance for electron-donating and electron-withdrawing aryl substituents on the thiol **83** and sulfoxonium ylide **14c** [91].

**Scheme 48:**
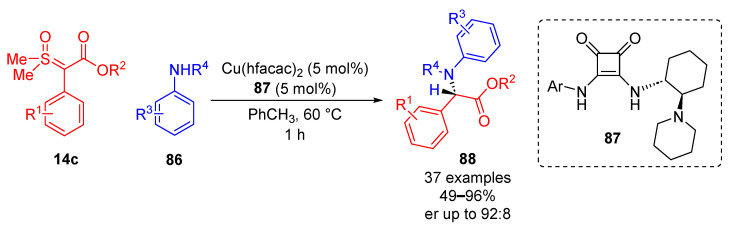
Burtoloso’s enantioselective N–H insertion of anilines by sulfoxonium ylides using cooperative copper–squaramide catalysis.

Following this, Burtoloso and colleagues reported the enantioselective N–H insertion of sulfoxonium ylides **14c** and anilines **86** using a copper–squaramide catalyst **87**, observing 37 substrate examples of the inserted product **88** with reaction yields of up to 96% and good enantiomeric ratios of up to 92:8 (Scheme 48) [92]. Anilines and sulfoxonium ylides possessing both EDG and EWG performed equally well when probed, indicating a broad tolerance of substrate types. The reaction conditions which gave the best results were in toluene for 1 h at 60 °C. The most effective catalytic system employed was a copper–squaramide complex, giving a high enantiomeric ratio and reaction yield. A series of phosphoric acids and thiourea catalysts were trialed, and they returned excellent reaction yields (>90%); however, they did not grant desirable enantiocontrol. The plausible reaction mechanism starts with the formation of a copper carbene as a result of the displacement of DMSO. The nucleophilic aniline attacks the central carbon, displacing the copper catalyst. The intermediate tautomerizes to form an enol, which complexes with the squaramide bound to copper and asymmetrically protonates the central carbon, allowing for the synthesis of the inserted product.

**Scheme 49:**
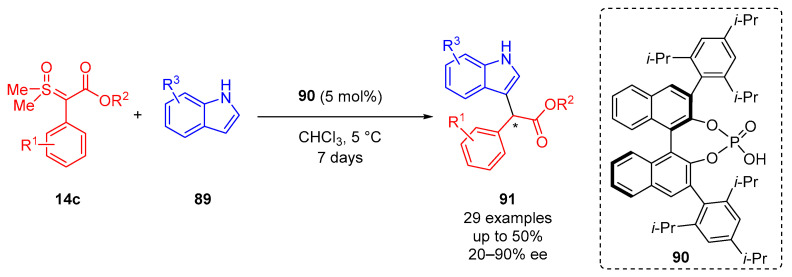
Burtoloso’s enantioselective indole insertion by sulfoxonium ylide using phosphoric acid catalysis.

Burtoloso and colleagues additionally reported the enantioselective C–H insertion of indoles **89** and *α*-aryl-*α*-estersulfoxonium ylides **14c** was promoted by phosphoric acid catalysis in 2021 (Scheme 49) [93]. In this case, a chiral phosphoric acid **90** (5 mol%) was employed in CHCl_3_ at 5 °C for a reaction duration of 7 days. Overall, 29 substrate examples were reported, giving a return of low to high enantiocontrol (20–93% ee) with moderate reaction yields of up to 50%. The proposed reaction mechanism starts with the initial protonation of the sulfoxonium ylide by the chiral phosphoric acid. The cationic sulfoxonium forms an ion pair with the anionic phosphate catalyst. The enantiocontrol stems from the chiral nature of the catalyst, allowing the indole to nucleophilically attack the carbon adjacent to the sulfoxonium group from the less sterically hindered side, displacing DMSO. The phosphoric acid catalyst regenerates by the deprotonation of a *β*-hydrogen.

**Scheme 50:**
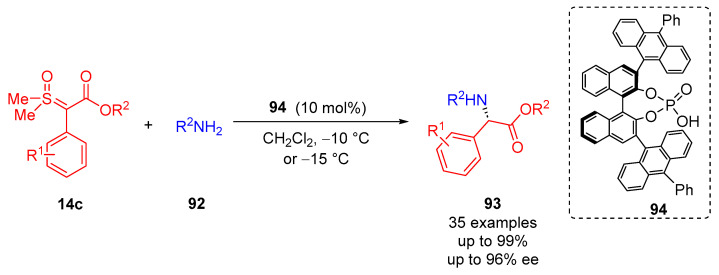
Sun’s chiral phosphoric acid-catalyzed asymmetric N–H insertion of amines and sulfoxonium ylides.

Similarly, Sun and colleagues introduced the asymmetric N–H insertion of amines **92** with sulfoxonium ylides **14c** through chiral phosphoric acid catalysis (**94**) for the synthesis of *α*-amino esters **93** (Scheme 50) **[94]**. In terms of substrate scope, 35 examples were reported with excellent reaction yields of up to 99% and excellent enantioselectivity of up to 96% ee. The reaction allowed broad tolerance for both activated and deactivated *α*-aryl-*α*-estersulfoxonium ylides, as well as the aryl amines that were explored. The reaction functioned most effectively at colder temperatures (−10 °C or −15 °C depending on the substrate) for long reaction times. CH_2_Cl_2_ was observed to be the best solvent when compared to CHCl_3_, which offered good reaction yields but moderate enantioselectivity. CH_3_CN, EtOAc and THF offered high reaction yields (up to 99%) and high enantioselectivity (up to 80% ee) at 25 °C. Moreover, 10 mol% of the chiral phosphoric acid **94** was observed to provide the desired results.

### 4.6. Miscellaneous Stereoselective Reactions of Sulfoxonium Ylides

*γ*-Lactones are structural elements that are found as part of many natural products (in about 10% of all natural products), including paraconic acids and numerous bicyclic and tricyclic ring systems (e.g., xanthatin) [95]. *γ*-Lactones have a range of biological activities, including strong antifungal, antibiotic, antitumor, antiviral and anti-inflammatory activities, which underscores their potential for pharmaceutical development [95,96,97]. *γ*-Lactones have also been used as building blocks for the synthesis of complex molecules [98,99].

**Scheme 51:**
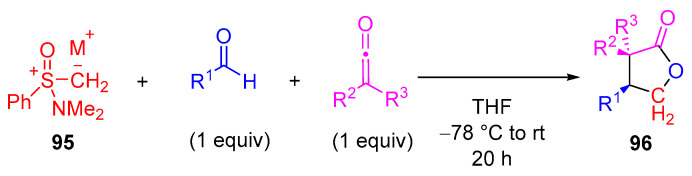
Kerrigan’s diastereoselective synthesis of *trans*-*γ*-lactones.

**Scheme 52:**
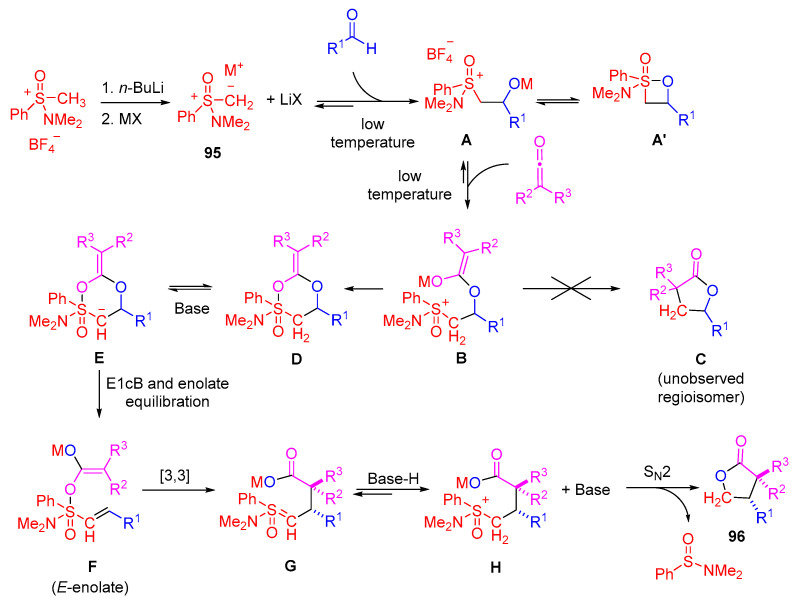
Proposed mechanism of Kerrigan’s diastereoselective synthesis of *trans*-*γ-*lactones.

Kerrigan’s group reported direct access to *trans*-*γ*-lactones **96** via an unprecedented extension to the Johnson−Corey−Chaykovsky reaction, in good yields (up to 93%) and with good diastereoselectivity (up to 92:8) (Scheme 51) (Table 8) [15,100,101,102,103]. They determined that chiral aminosulfoxonium ylide **95**, obtained through treatment of salt with base, was superior to all other onium ylides for *γ*-lactone formation. In all cases, tetrafluoroborate (BF_4_^−^) was used as a non-nucleophilic counterion for sulfoxonium salt /ylide **95** in order to minimize side reactions with ketenes (e.g., with iodide as a counterion). This methodology was found to be versatile enough to tolerate ketenes of quite different reactivity, including dimethylketene, alkylarylketenes and even diphenylketene. Furthermore, the level of diastereoselectivity was found to be good for *ortho*-substituted aromatic aldehydes, heteroaromatic substituted aldehydes and the *α*-branched aliphatic aldehyde, isobutyraldehyde. Generally, the best yields of *γ*-lactone were obtained with aromatic aldehydes. The lower yields for the aliphatic aldehyde-derived lactones may be attributed to side reactions involving enolization of starting materials and intermediates. It is speculated that the additive MgCl_2_ has a number of roles in the reaction system. Primarily, it activates aliphatic aldehydes, such as isobutyraldehyde, to undergo reaction with the sulfoxonium ylide **95**. A second possible effect is that, under certain conditions, it may enable greater organization (through chelation) in the transition state for [3,3]-sigmatropic rearrangement of **F** to **G** (Scheme 52).

In the proposed mechanism for the reaction (Scheme 52), ylide **95** is added to aldehyde to give betaine **A**, most likely in equilibrium with oxathietane **A′** [104,105,106]. Reaction of betaine **A** with ketene gives rise to enolate **B**, which is formed stereoselectively as an *E*-enolate (for M = Li) at this point. Deprotonation of **D** by a base (e.g., ylide **95**) gives sulfurane oxide anion **E**, which then undergoes E1cB elimination to give enolate **F** [107]. Enolate **F** then undergoes a [3,3]-sigmatropic rearrangement in stereoselective fashion, presumably via a chair transition state, to afford carboxylate **G [108]**. Protonation of **G** gives **H**, which undergoes intramolecular S_N_2 (5-*exo-tet*) cyclization to provide *trans*-*γ*-lactone **96**.

They reasoned that *γ*-lactones could be formed in enantioselective fashion, provided a suitable enantioenriched aminosulfoxonium salt could be identified [109]. Chiral aminosulfoxonium salt was obtained through a four-step procedure from commercially available methylphenyl sulfoxide [110,111,112,113]. Enantioenriched aminosulfoxonium (*S*)-ylide **97** was subjected to reaction with a variety of aldehydes (both aromatic and aliphatic) and disubstituted ketenes, leading to the formation of *α*,*β*-substituted *γ*-lactones **96** in moderate to very good diastereoselectivity (dr up to 95:5) and with enantiomeric excesses of up to 79% ee (Table 9). The best levels of enantioselectivity were observed in the reactions using with isobutyraldehyde and various alkylarylketenes.

**Scheme 53:**
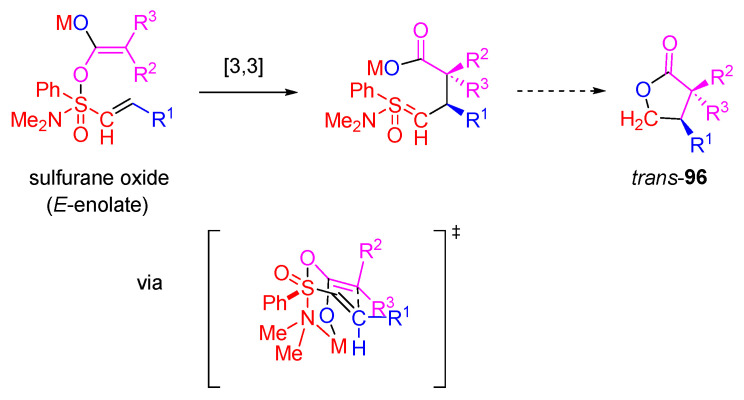
Proposed transition state for [3,3]-rearrangement of intermediate sulfurane oxide.

Diastereoselectivity in *γ*-lactone forming reaction may be rationalized by a [3,3]-sigmatropic rearrangement of sulfurane oxide **E**, involving a six-membered chair-like transition state, where the sterically bulkier substituents (R^1^ and R^3^) preferentially occupy pseudoequatorial positions (Scheme 53) [110,111,112,114]. They propose that the O-enolate and –NMe_2_ substituents occupy apical positions at sulfur (in a trigonal bipyramidal geometry), which is consistent with the reported arrangement of cyclic sulfurane oxides bearing electronegative substituents [105,107,115,116]. High enantioselectivity may be achieved through the rigid transition state depicted in Scheme 53, where the phenyl substituent blocks the approach by the enolate moiety to the *re* face of the *α*,*β*-unsaturated sulfurane oxide [117,118].

**Scheme 54:**
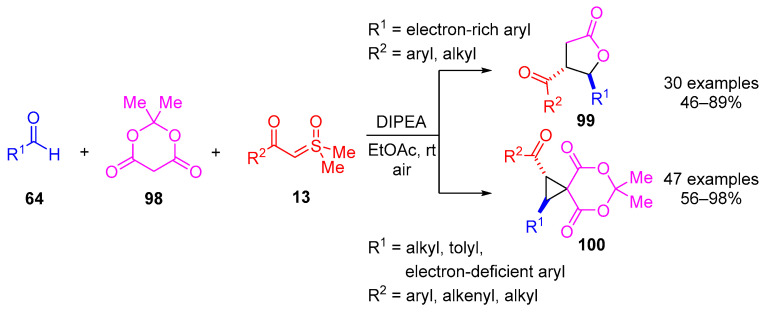
Peng’s diastereoselective synthesis of *trans*-4,5-disubstituted *γ*-butyrolactones from aldehydes, sulfoxonium ylides and Meldrum’s acid, using DIPEA.

In 2021, Peng and colleagues reported the synthesis of 4,5-disubstituted *γ*-butyrolactones **99** from aldehydes **64**, *α*-carbonylsulfoxonium ylides **13** and Meldrum’s acid **98** using *N*,*N*-diisopropylethylamine (DIPEA) (Scheme 54) [119]. The reaction displayed diastereoselectivity for *trans*-*γ*-butyrolactones **99**, which were formed in moderate to excellent yields (30 examples, 46–98%). They discovered that the reaction provided *trans*-spirocyclopropanes **100** from electron-deficient benzaldehydes (or methyl-substituted benzaldehydes) with alkyl or aryl-substituted *β*-ketosulfoxonium ylides in moderate to excellent yields (47 examples, 56–98%). However, the formation of *trans*-*γ*-butyrolactones was realised through the reaction involving electron-rich benzaldehydes and alkyl- or aryl-substituted *β*-ketosulfoxonium ylides.

**Scheme 55:**
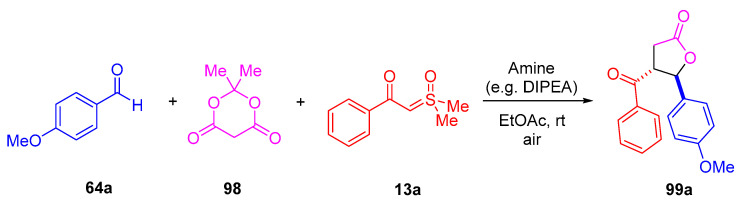
Synthesis of 4-benzoyl-5-(4-methoxyphenyl)-*γ*-butyrolactone.

The reaction was optimized by focusing on the synthesis of 4-benzoyl-5-(4-methoxyphenyl)-*γ*-butyrolactone **99a** from 4-methoxybenzaldehyde **64a**, *β*-benzoyl-substituted dimethylsulfoxonium methylide **13a** and Meldrum’s acid **98** (Scheme 55). The reaction proceeded well in CH_2_Cl_2_ (73%) and moderately well when attempted in CCl_4_ (32%). However, no reaction was observed when examined in EtOH. The most effective reaction conditions were noted as EtOAc (98%) employing 2 equivalents of DIPEA, 1.5 equivalents of aldehyde **64**, 2 equivalents of Meldrum’s acid **98** and 1 equivalent of sulfoxonium ylide **13a**. An excellent yield of 94% was observed when triisopropylamine (instead of DIPEA) was tested. Moderate to excellent yields were observed when using triethylamine (32–86%), while 4-dimethylaminopyridine provided a yield of 41%.

**Scheme 56:**
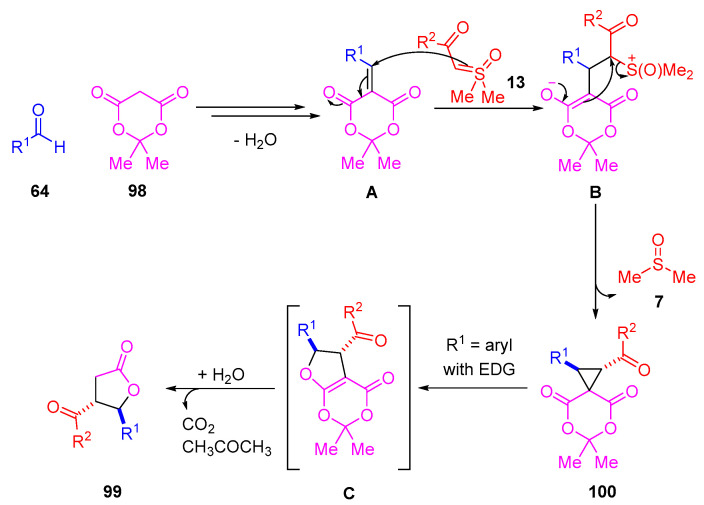
Proposed reaction mechanism for Peng’s diastereoselective synthesis of *trans*-4,5-disubstituted *γ*-butyrolactones.

The proposed reaction mechanism for the synthesis of *trans*-*γ*-butyrolactones and *trans*-spirocyclopropane proceeds through a similar pathway (Scheme 56, Table 10). The proposed reaction mechanism starts with an initial Knoevenagel condensation of an aldehyde **64** with Meldrum’s acid **98**, which forms an alkylidene/arylidene intermediate **A**. A sulfoxonium ylide **13** undergoes a 1,4-addition and the resulting enolate of intermediate **B** displaces DMSO **7**, leading to the formation of cyclopropane product **100**. In cases where electron-rich benzaldehydes are examined, the *trans*-spirocyclopropane undergoes a Cloke-Wilson rearrangement to form a dihydrofuran intermediate **C** [120,121,122]. Aided by water, **C** decarboxylates and liberates acetone to form *trans*-*γ*-butyrolactone **99** (Scheme 56).

**Scheme 57:**
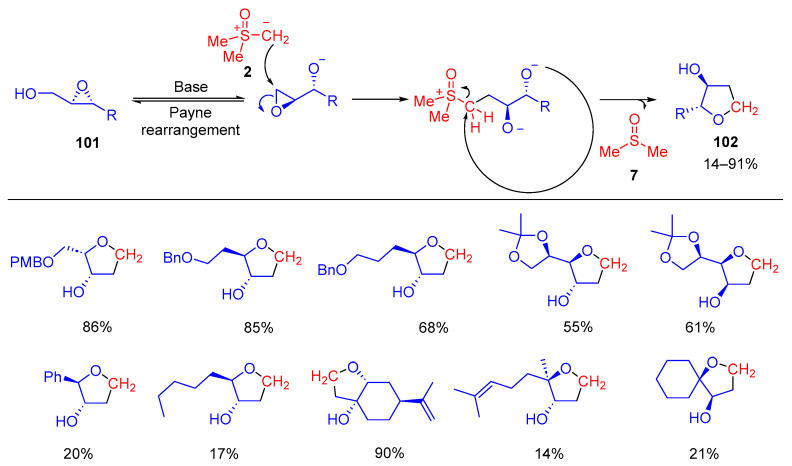
Borhan’s synthesis of diastereomerically and enantiomerically pure 2,3-disubstituted tetrahydrofurans.

Borhan and co-workers introduced a methodology for regioselective nucleophilic substitution reactions of enantiopure 2,3-epoxy alcohols **101** with dimethylsulfoxonium methylide **2** to yield diastereomerically and/or enantiomerically pure disubstituted tetrahydrofuran rings **102** (Scheme 57) [123]. In this methodology, the stereochemistry that is set by the asymmetric epoxidation is translated fully to the final product. This approach provided an efficient access to tetrahydrofuran rings with stereodefined substituents from chiral 2,3-epoxy-alcohols **101** which are relatively simpler than the method via the Sharpless asymmetric epoxidation reaction.

**Scheme 58:**
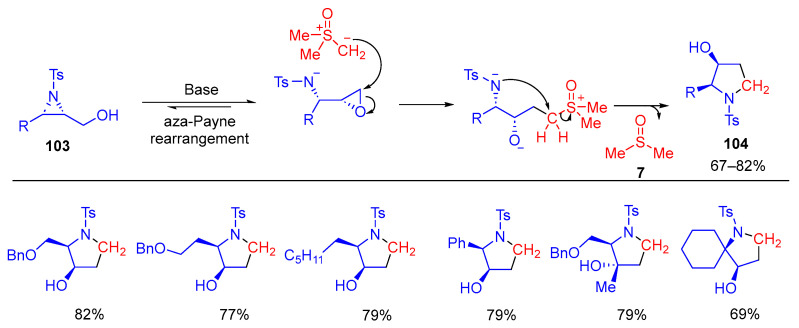
Borhan’s diastereoselective synthesis of substituted pyrrolidines **104** from 2,3-aziridin-1-ols **103**.

The same group further extended this methodology to the sulfoxonium ylide-based aza-Payne rearrangement of 2,3-aziridin-1-ols **103** for efficient diastereoselective synthesis of substituted pyrrolidines **104** (Scheme 58) **[124]**. The aza-Payne rearrangement under basic reaction conditions favors the formation of epoxy amides. Subsequent nucleophilic attack of the epoxide by the sulfoxonium ylide generates a dianion intermediate, which upon a 5-*exo-tet* ring-closure yields the desired pyrrolidine **104**, thus completing the relay of the three-membered ring to the five-membered nitrogen-containing ring system. The stereochemistry present in the enantioenriched aziridinols is translated fully to the final products.

**Scheme 59:**
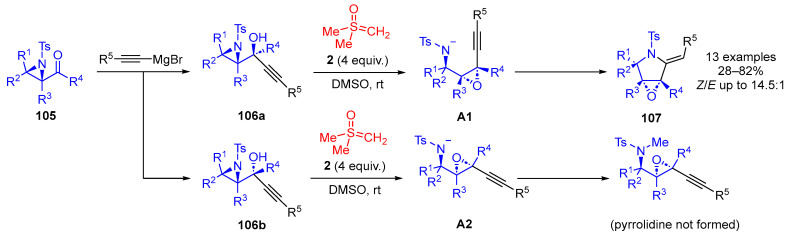
Borhan’s diastereoselective aza-Payne/hydroamination of chiral aziridinol.

They also reported a sulfoxonium ylide-mediated facile tandem aza-Payne/hydroamination reaction of chiral *syn*-aziridinol **106** for diastereoselective synthesis of highly functionalised pyrrolidines **107** (Scheme 59) [125]. Addition of alkynyl Grignard reagents to chiral 2,3-aziridinals **105** predominantly yielded *syn*-aziridinols **106**. Treatment of this chiral *syn*-aziridinol **106a** with sulfoxonium ylide **2** led initially to an aza-Payne rearrangement, which juxtaposed the amine and the alkyne in a favorable orientation **A1** to complete the hydroamination. The authors also reported that the *anti*-aziridinol **106b** underwent the aza-Payne rearrangement but could not proceed further with the hydroamination.

**Scheme 60:**
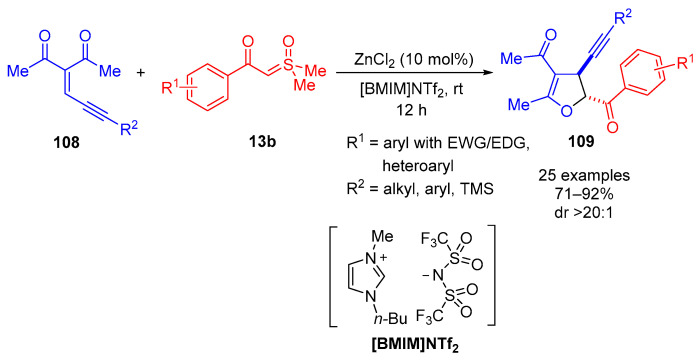
Wu’s diastereoselective synthesis of 2,3-dihydrofurans from *α*-ketosulfoxonium ylides and enynones.

**Scheme 61:**
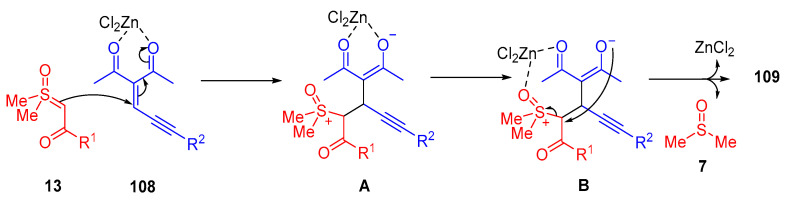
Proposed reaction mechanism of Wu’s Lewis acid-catalyzed synthesis of 2,3-dihydrofurans.

In 2020, Wu and colleagues reported a diastereoselective synthesis of 2,3-dihydrofurans **109** from *α*-ketosulfoxonium ylides **13b** and enynones **108** induced by Lewis acid-catalysis in ionic liquids (Scheme 60) [126]. The proposed mechanism proceeds through the Lewis acid activation of enynone **108**, which allows the conjugate addition of *α*-ketosulfoxonium ylide **13b** to form intermediate **A** (Scheme 61). Cyclization (5-*exo-tet*) of the enolate’s O-atom onto the carbon adjacent to the sulfoxonium group of **B**, results in the formation of the 2,3-dihydrofuran product **109**. They reported 25 product examples with good to excellent yields of up to 92% and excellent diastereoselectivity (dr > 20:1) [126].

**Scheme 62:**
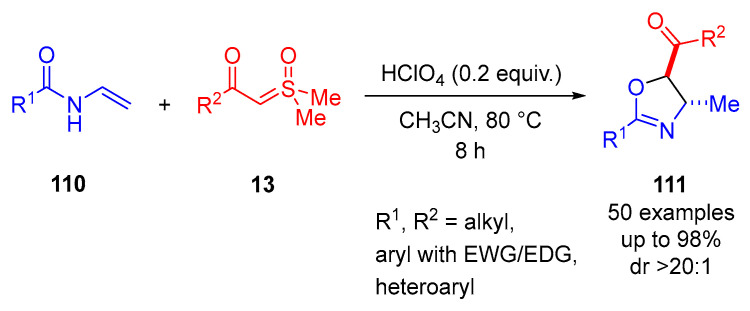
Huang’s diastereoselective synthesis of *trans*-oxazolines using Brønsted acid-catalysis.

Huang and colleagues reported a metal-free diastereoselective synthesis of trisubstituted *trans*-oxazolines **111** from sulfoxonium ylides **13** and enamides **110** by Brønsted acid-catalysis (Scheme 62) [127]. The preferred catalyst was perchloric acid (0.2 equivalents) in CH_3_CN at 80 °C for 8 h. A broad substrate scope was explored and resulted in 50 examples with reaction yields of up 98% and excellent diastereoselectivity (dr >20:1). The reaction demonstrated tolerance for alkyl, heteroaryl and EWG/EDG-substituted aryl groups.

**Scheme 63:**
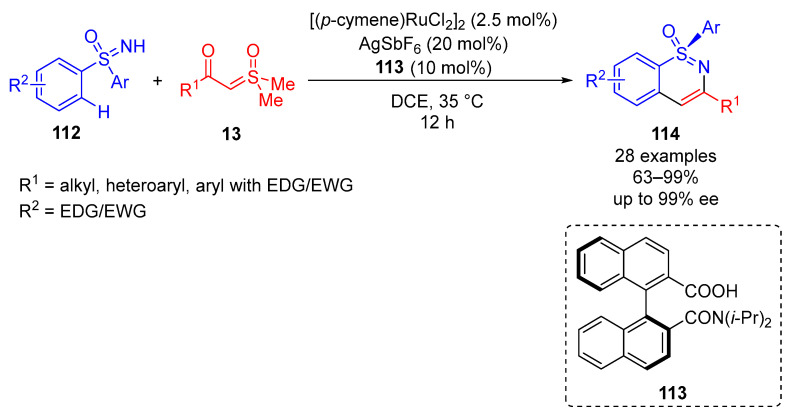
Shi’s enantioselective synthesis of stereogenic sulfur-containing sulfoximines through carboxylic acid-catalysis.

In 2021, Shi and colleagues reported a ruthenium-catalyzed enantioselective synthesis of stereogenic sulfur-containing sulfoximines **114** through the annulation of aryl sulfoximines **112** and *α*-ketosulfoxonium ylides **13** (Scheme 63) [128]. In total, 28 examples of stereogenic sulfur-containing sulfoximines **114** were presented in good to excellent yields (63–99%) and with excellent enantioselectivity (up to 99% ee). The reaction performed best in DCE, at 35 °C for 12 h when using 2.5 mol% of [(*p*-cymene)RuCl_2_]_2_, 20 mol% of AgSbF_6_ and 10 mol% of the chiral binaphthyl monocarboxylic acid ligand **113**. The method exhibited substrate tolerance for activated and deactivated aryl sulfoximines **112** and a tolerance for alkyl, heteroaryl and EWG/EDG-substituted aryls in the R^1^ position.

**Scheme 64:**
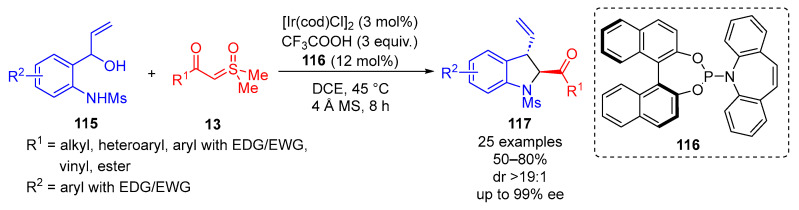
Sun’s diastereo- and enantioselective synthesis of 3-vinyl indolines from sulfoxonium ylides and hydroxyallyl anilines.

In 2023, Sun and colleagues reported an iridium-catalyzed asymmetric [4 + 1] cycloaddition of *α-*carbonylsulfoxonium ylides **13** and hydroxyallylanilines **115** resulting in 3-vinylindolines **117** (Scheme 64) [129]. The reaction used 3 mol% of [Ir(cod)Cl]_2_, three equivalents of TFA and 12 mol% of Carreira’s ligand **116** [130,131]. The preferred reactions conditions reported the use of DCE as solvent at 45 °C for 8 h, utilizing molecular sieves (4 Å). A broad substrate scope was tolerated when probed under the optimal conditions, which returned 25 examples of moderate to very good yields (50–80%) with excellent enantio- and diastereoselectivity (up to 99% ee and dr > 19:1). Electron-rich and electron-deficient hydroxylallylanilines were tolerated when EWG/EDG were trialed in the R^2^ position, and vinyl, ester, alkyl, heteroaryl and EWG/EDG-substituted aryls returned favorable results when probed in the R^1^ position.

## 5. Conclusions

Sulfoxonium ylides are an intriguing and highly valuable class of zwitterionic intermediates, gaining significant attention in organic synthesis due to their ability to facilitate the formation of cyclic products as well as inserted products. Since their synthetic capabilities were discovered in the 1960s in the formation of monocyclic compounds (e.g., epoxides, cyclopropanes and aziridines), further studies have been conducted in exploring their potential to carry out stereoselective reactions. In recent times, through interrupted Johnson–Corey–Chaykovsky reaction strategies, sulfoxonium ylides have been shown to enable the stereoselective synthesis of polycyclic epoxide- and cyclopropane-based structures (e.g., 2,3-epoxyhexahydrofluoren-9-ones, cyclopropane-fused tetralones, cyclopropane-fused indanes and indeno spirocyclopropanes). However, the synthesis of aziridine-, cyclobutane-, oxetane- and azetidine-based polycyclic structures through stereoselective interrupted Johnson–Corey–Chaykovsky reaction strategies using sulfoxonium ylides remains unexplored. The rapidly developing area of olefination reactions has seen great success in recent years to synthesize both *E-* and *Z-*isomers selectively using a range of catalytic strategies, such as through the use of palladium-catalysis, iridium-catalysis or carbene-mediated transition metal catalysis. Sulfoxonium ylides’ ability to perform stereoselective insertion reactions has also been successfully explored through the insertion of N–H bonds, S–H bonds and C–H bonds. Indeed, chiral phosphoric acid-catalysis and thiourea-catalysis has been shown to be effective in inducing enantiocontrol. However, the stereoselective insertion of the related P–H bonds remains largely unexplored. In addition, the versatility of sulfoxonium ylides in synthesizing five-membered rings (e.g., *γ-*lactones, tetrahydrofurans, pyrrolidines, 2,3-dihydrofurans and oxazolines) and aryl-fused heterocycles (e.g., stereogenic sulfur-containing sulfoximines and indolines) has been successfully demonstrated. The development of enantioselective photocatalytic reactions involving sulfoxonium ylides remains a largely untapped area and is poised for future breakthroughs.

## Figures and Tables

**Table 1:** Selection of results from Ramasastry’s diastereoselective epoxidation.

** 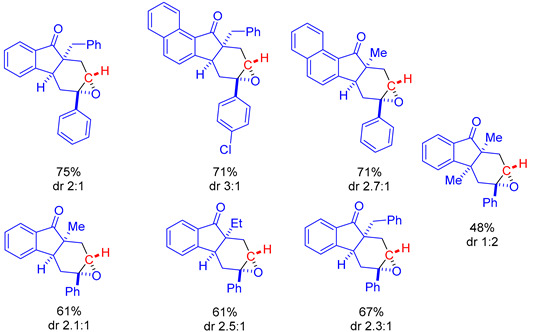 **

**Table 2:** Selected results from Huang’s asymmetric synthesis of trifluoromethylated aziridines.

** 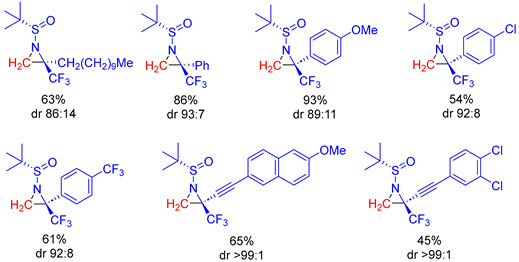 **

**Table 3:** Diastereoselective synthesis of aziridines from chiral *N*-tert-butanesulfinyl ketimino esters and sulfoxonium ylides.

** 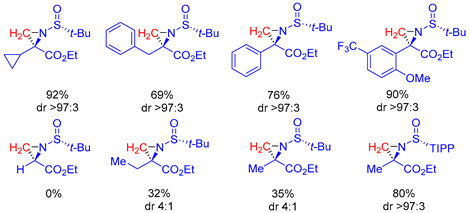 **

**Table 4:** Selection of results from Li’s diastereoselective synthesis of chiral diketo aziridines.

** 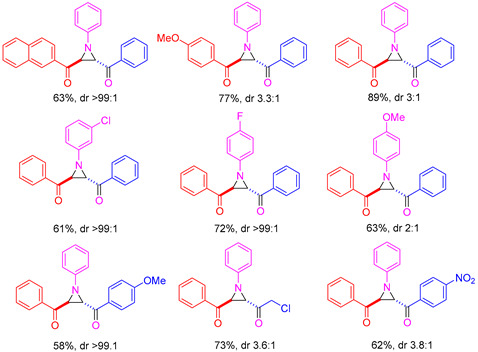 **

**Table 5:** Selection of *Z*-olefins formed from Maulide’s cross-olefination.

** 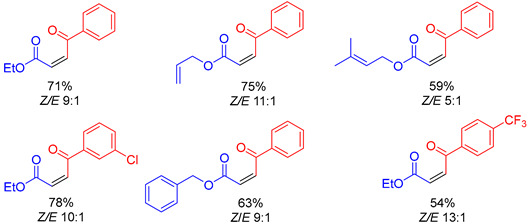 **

**Table 6:** Selection of Jiang’s stereoselective olefination reaction results.

** 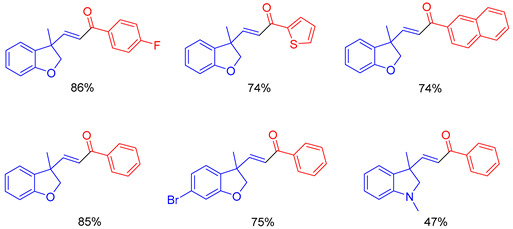 **

**Table 7:** Reaction results of Liao’s diastereoselective synthesis of disubstituted alkenes using iridium-catalysis.

** 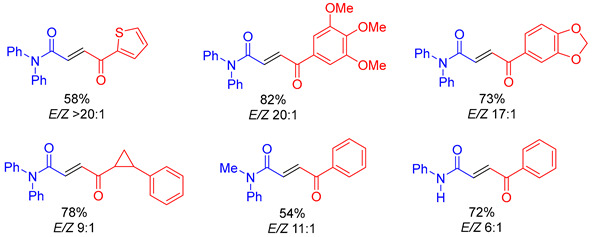 **

**Table 8:** Selection of *trans-γ-*lactones from Kerrigan’s diastereoselective synthesis.

** 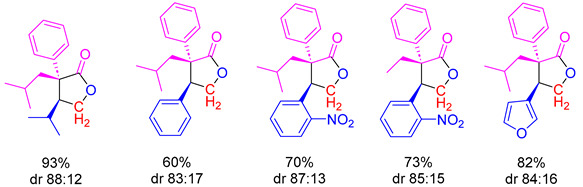 **

**Table 9:** Kerrigan’s asymmetric synthesis of *γ*-lactones.

** 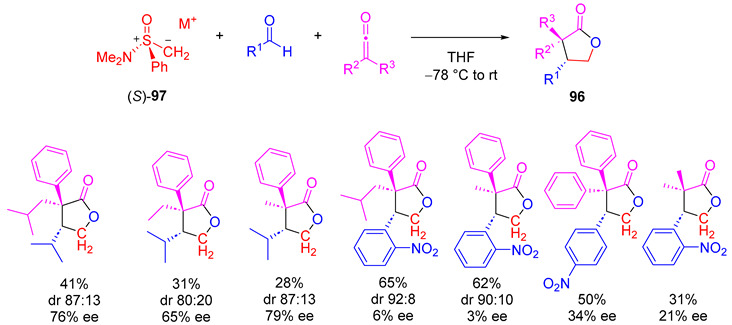 **

**Table 10:** Representative reaction results.

** 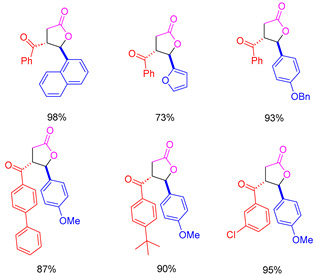 **
